# Mesomeric Acceleration Counters Slow Initiation of Ruthenium–CAAC Catalysts for Olefin Metathesis (CAAC = Cyclic (Alkyl)(Amino) Carbene)

**DOI:** 10.1021/acscatal.2c03828

**Published:** 2023-04-05

**Authors:** Xinrui Ou, Giovanni Occhipinti, Eliza-Jayne Y. Boisvert, Vidar R. Jensen, Deryn E. Fogg

**Affiliations:** † Center for Catalysis Research & Innovation, and Department of Chemistry and Biomolecular Sciences, University of Ottawa, Ottawa, Ontario K1N 6N5, Canada; ‡ Department of Chemistry, University of Bergen, Allégaten 41, N-5007 Bergen, Norway

**Keywords:** olefin metathesis, initiation, inductive, mesomeric, cyclic (alkyl)(amino) carbene, productivity

## Abstract

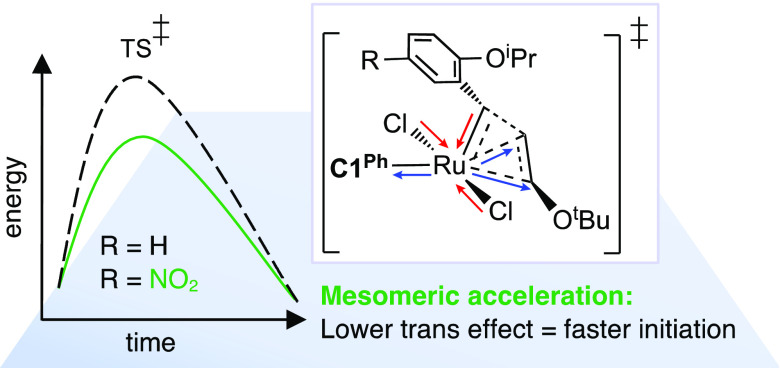

Ruthenium catalysts bearing cyclic (alkyl)(amino)carbene (CAAC) ligands can attain very high productivities in olefin metathesis, owing to their resistance to unimolecular decomposition. Because the propagating methylidene species RuCl_2_(CAAC)(=CH_2_) is extremely susceptible to bimolecular decomposition, however, turnover numbers in the metathesis of terminal olefins are highly sensitive to catalyst concentration, and hence loadings. Understanding how, why, and how rapidly the CAAC complexes partition between the precatalyst and the active species is thus critical. Examined in a dual experimental–computational study are the rates and basis of initiation for phosphine-free catalysts containing the leading CAAC ligand **C1**^**Ph**^, in which a CMePh group α to the carbene carbon helps retard degradation. The Hoveyda-class complex **HC1**^**Ph**^ (RuCl_2_(L)(=CHAr), where L = **C1**^**Ph**^, Ar = C_6_H_3_-2-O^*i*^Pr-5-R; R = H) is compared with its nitro-Grela analogue (**nG-C1**^**Ph**^; R = NO_2_) and the classic Hoveyda catalyst **HII** (L = H_2_IMes; R = H). *t*-Butyl vinyl ether (*t*BuVE) was employed as substrate, to probe the reactivity of these catalysts toward olefins of realistic bulk. Initiation is ca. 100× slower for **HC1**^**Ph**^ than **HII** in C_6_D_6_, or 44× slower in CDCl_3_. The rate-limiting step for the CAAC catalyst is cycloaddition; for **HII**, it is *t*BuVE binding. Initiation is 10–13× faster for **nG-C1**^**Ph**^ than **HC1**^**Ph**^ in either solvent. DFT analysis reveals that this rate acceleration originates in an overlooked role of the nitro group. Rather than weakening the Ru–ether bond, as widely presumed, the NO_2_ group accelerates the ensuing, rate-limiting cycloaddition step. Faster reaction is caused by long-range mesomeric effects that modulate key bond orders and Ru-ligand distances, and thereby reduce the trans effect between the carbene and the trans-bound alkene in the transition state for cycloaddition. Mesomeric acceleration may plausibly be introduced via any of the ligands present, and hence offers a powerful, tunable control element for catalyst design.

## Introduction

**Chart 1:**
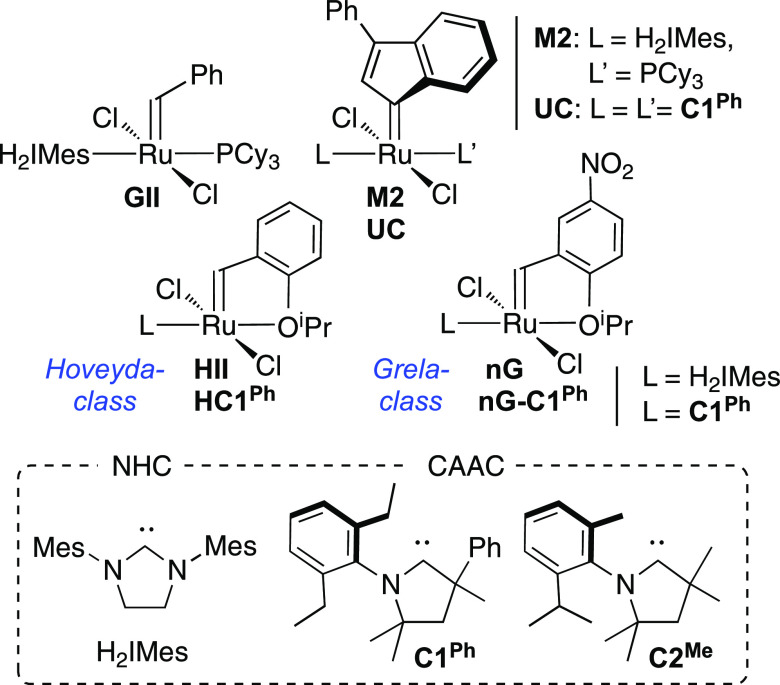
Metathesis Catalysts and Carbene Ligands Discussed

Ruthenium–carbene catalysts have transformed the scope and synthetic power of olefin metathesis.^1,2^ A step-change in reactivity was enabled by the discovery of Grubbs-class complexes bearing a strongly donating N-heterocyclic carbene (NHC) ligand (e.g., **GII**, Chart 1).^3^ As evidence accumulated that the stabilizing PCy_3_ ligand limits both reaction rates^4−6^ and catalyst longevity,^7^ attention turned to phosphine-free catalysts such as the Hoveyda and nitro-Grela catalysts (**HII** and **nG**, respectively),^8−11^ particularly in contexts such as pharmaceutical manufacturing, where throughput and high turnover numbers (TONs) are paramount.^12^

This leading position is now being contested by new ruthenium catalysts stabilized by cyclic (alkyl)(amino) carbene (CAAC) ligands.^13,14^ The CAAC catalysts have enabled breakthrough performance in topical current applications of olefin metathesis, including the synthesis of macrocycles via ring-closing metathesis (mRCM),^15,16^ and the transformation of renewable internal olefins into 1-olefins via cross-metathesis with ethylene (“ethenolysis”).^16−20^ Catalysts bearing the phenyl-protected **C1**^**Ph**^ ligand (Chart 1) stand out for their resistance to degradation by nucleophiles or Bronsted base,^21^ water,^22,23^ and unidentified contaminants in technical-grade ethylene,^16^ while a **C2**^**Me**^ derivative enabled TONs up to 340,000 in ethenolysis with ultra-high-purity ethylene at 1 ppm catalyst.^17^ All, however, are highly sensitive to catalyst concentration.^17,22,24−26^ Indeed, a ca. 50% drop in TONs is evident in the Ru-**C2**^**Me**^ ethenolysis study when catalyst loadings were increased from 1 to just 3 ppm.^17^

Given that bimolecular decomposition is mediated by the four-coordinate active species,^24,27^ these findings call new attention to the rates at which the catalysts enter the active cycle. To date, studies of initiation for the CAAC catalysts have focused on detailed comparisons of different CAAC ligands within the Hoveyda catalyst family,^17^ or of catalysts that differ in both carbene and catalyst architecture (for example, comparison of the Grela-class catalyst **nG-C1**^**Ph**^ with indenylidene catalysts, including **UC** and **M2**; Chart 1).^16^ Noteworthy in the latter case is the dramatically faster initiation (ca. 900×) established for **nG-C1**^**Ph**^ relative to the H_2_IMes indenylidene catalyst **M2**, underscoring the point that entry into the active cycle is controlled by both catalyst architecture and ligand properties. As this example also illustrates, however, differences in the catalyst platform hamper a systematic comparison of leading CAAC and NHC ligands based on literature data. Assessment of directly analogous catalysts for the important **C1**^**Ph**^ and H_2_IMes systems is highly desirable as a step toward understanding the relationship between rates of initiation and productive metathesis,^28,29^ vs bimolecular decomposition.^24,27^

Here we set out to deconvolute the impact on initiation rates of the carbene ligand (H_2_IMes vs **C1**^**Ph**^) and the benzylidene substituent within the dominant phosphine-free catalyst platforms: that is, catalysts of the Hoveyda and nitro-Grela classes (Chart 1). These precatalysts are stabilized by a “placeholder” ether ligand that is lost on entry to the active cycle. In the more reactive nitro-Grela variants, the electron-withdrawing nitro substituent *para* to the oxygen donor accelerates initiation^10,11^ (in principle by weakening the Ru–oxygen bond,^29−32^ a widely accepted premise reexamined herein). A combined experimental and density functional theory (DFT) analysis reveals that the CAAC ligand **C1**^**Ph**^ retards initiation by up to ca. 100× for **nG-C1**^**Ph**^ vs **HII**, but that incorporating a nitro-benzylidene group limits this impact to a 10-fold decrease. Unexpectedly, the impact of the NO_2_ group is shown to arise from long-distance mesomeric effects that accelerate the bond-breaking and bond-making processes central to olefin metathesis, a finding with important implications for catalyst design and performance.

## Results and Discussion

### Assessing Initiation Rates

**Figure 1:**
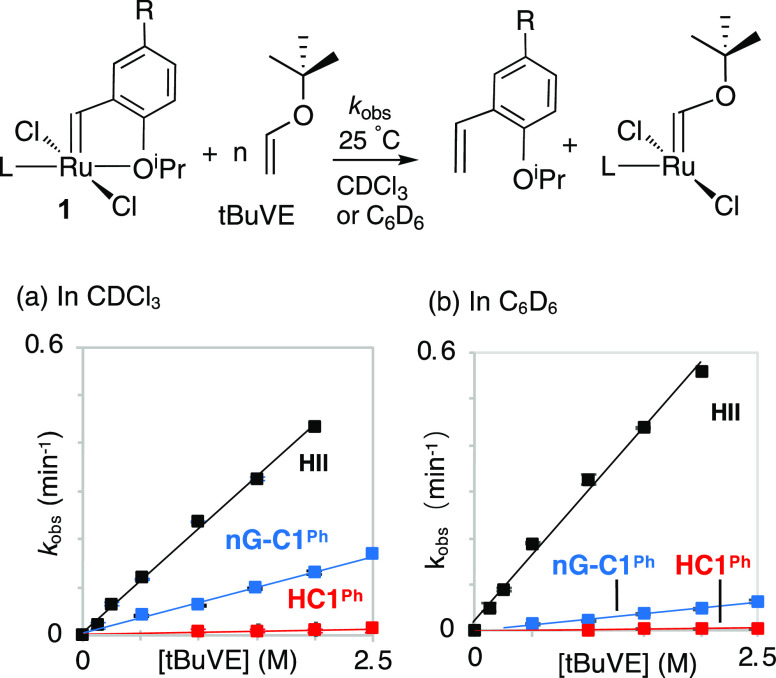
Extracting second-order rate constants for the metathesis of **1** (a collective designation for the Hoveyda- and Grela-class precatalysts; R = H or NO_2_, respectively) with *t*BuVE at 25 °C; *k*_obs_ = *k*_1_[*t*BuVE]. (a) In CDCl_3_. (b) In C_6_D_6_. For rate curves and kinetics plots, see Figures S2–S8.

To benchmark rates of initiation for the CAAC complexes **HC1**^**Ph**^ and **nG-C1**^**Ph**^ against H_2_IMes catalyst **HII**, we undertook reaction with *t*-butyl vinyl ether (*t*BuVE) at 25 °C. This bulky substrate was chosen to probe catalyst reactivity toward sterically demanding substrates, a question relevant to the metathesis of 1-olefins bearing bulky homoallylic substituents (commonplace in target-directed synthesis), and metathesis of sterically encumbered internal olefins. Cross-metathesis with vinyl ethers is a convenient, widely used means of assessing initiation of Ru metathesis catalysts. Because reaction is rapid and irreversible, and the Ru products are metathesis-inactive Fischer carbenes (Figure 1),^4,33,34^ initiation rates are unperturbed by propagation or recapture of the styrenyl ether.

Loss of the alkylidene signal for the precatalysts **1** was monitored by ^1^H NMR analysis at 25 °C in CDCl_3_. A linear dependence of *k*_obs_ on the concentration of *t*BuVE was evident over a wide concentration range (e.g., 0.5–2.5 M, in the case of **nG-C1**^**Ph**^),^35,36^ despite the steric bulk of *t*BuVE. This observation is consistent with prior findings that reaction rates depend on alkene concentrations for phosphine-free catalysts.^37−40^ The rate difference is in excellent agreement with that reported for the H_2_IMes analogues: specifically, **nG** was reported to initiate 12× faster than **HII** in CH_2_Cl_2_ at 25 °C.^41^ Also evident from Table 1 is the faster initiation conferred by the H_2_IMes ligand relative to **C1**^**Ph**^, with **HII** initiating ca. 40× faster than **HC1**^**Ph**^ in CDCl_3_.^42^

**Table 1:** Measured Initiation Rates^a^

complex	solvent	*k*_1_ (M^–1^ s^–1^)	*k*_rel_^b^
**HII**	CDCl_3_	36.2 ± 0.7 × 10^–4^	44
**nG-C1**^**Ph**^	CDCl_3_	10.7 ± 0.5 × 10^–4^	13
**HC1**^**Ph**^	CDCl_3_	0.83 ± 0.03 × 10^–4^	1
**HII**	C_6_D_6_	45 ± 1 × 10^–4^	105
**nG-C1**^**Ph**^	C_6_D_6_	4.1 ± 0.1 × 10^–4^	9.5
**HC1**^**Ph**^	C_6_D_6_	0.43 ± 0.01 × 10^–4^	1

a Conditions as in Figure 1.

b *k*_rel_ = *k*_1_ normalized to the value for **HC1**^**Ph**^, the slowest system, in each solvent.

The general trend **HII** > **nG-C1**^**Ph**^ > **HC1**^**Ph**^ is maintained in C_6_D_6_, but an intriguing solvent-dependence is apparent for the **C1**^**Ph**^ derivatives, for which rates are approximately half those measured in CDCl_3_. **HII** is little affected in comparison, initiating slightly faster in C_6_D_6_. The rate difference between **HII** and **HC1**^**Ph**^ is consequently amplified in benzene, with **HII** initiating ca. 100× faster. The difference between **nG-C1**^**Ph**^ and **HC1**^**Ph**^, however, is slightly compressed (dropping from 13- to 10-fold). It may be noted that the H_2_IMes analogues **nG** and **HII** were also found to show surprisingly comparable initiation rates in toluene: **nG** was reported to react only 2–3× faster than **HII**, over temperatures ranging from 10–40 °C.^29,34,43^

In short, differences between the Hoveyda and Grela platforms are considerably smaller than differences resulting from the nature of the carbene. Although a steric contribution to the latter cannot be ruled out, we suggest that the dominant effect is the greatly increased trans effect/trans influence of the CAAC ligand,^24,26^ a consequence of its stronger σ-donor and π-acceptor properties.^14^ We recently demonstrated that the high trans influence of the CAAC ligand plays a pivotal role in the decomposition of Ru–CAAC metathesis catalysts.^24,26^ In the present context, it is expected to weaken the trans-disposed Ru–O bonds in **HC1**^**Ph**^ and **nG-C1**^**Ph**^, but the anticipated rate acceleration appears to be offset by other reaction steps. We undertook computational analysis to explore the relevant elementary steps, and their impact on rates of initiation.

### Computational Evaluation of the Initiation Barriers

**Figure 2:**
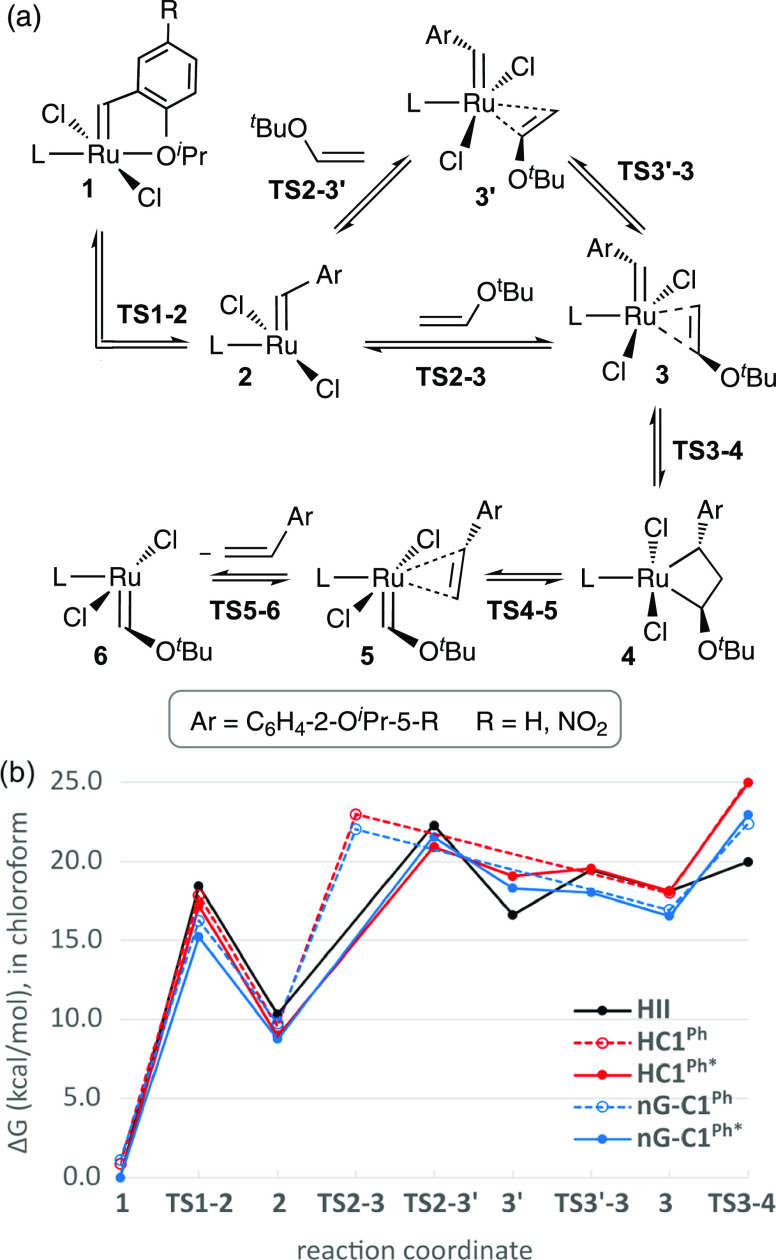
(a) Elementary steps in the reaction of **1** with *t*BuVE. (b) Calculated Gibbs free energies for key stationary points relative to the most stable rotamer of **1** in CHCl_3_. Only the most favorable pathways are shown. For numerical values, see Table S3. Within the **C1**^**P**h^ species, (*) denotes the rotamer in which the alkylidene is syn to CMePh; that in which it is syn to N–Ar bears no asterisk (see Figure S9). In **3** and **3′**, the *t*BuVE C=C bond is oriented essentially parallel to or orthogonal to the Ru=C alkylidene bond, respectively.

Metathesis of vinyl ethers is irreversible, as noted above, and prior DFT studies of the pathway show a sharp drop in free energy commencing at an early stage, during cycloaddition to form the metallacyclobutane **4**.^37,44^ This drop is due to the exceptional stability of Fischer carbene **6**, the ruthenium product of cycloreversion (see Figure S20; SI Section S2.3.1). Our calculations confirm that this stability is maintained even for the sterically demanding vinyl ether *t*BuVE. In consequence, dissociation of the alkene product is relatively fast (Figure S20), and the rate-determining step for initiation precedes **4**. As shown in Figure 2, the rate-determining step in initiation of **HII** with *t*BuVE is substrate binding to 14-electron complex **2** (via **TS2-3′**), whereas for the CAAC catalysts it is cycloaddition (via **TS3-4**). Initiation barriers reported here are hence calculated as the free-energy difference between precatalyst **1** and transition state **TS2-3′** (for **HII**), or between **1** and transition state **TS3-4** (for **HC1**^**Ph**^ and **nG-C1**^**Ph**^).

Olefin binding has been reported to occur via an interchange mechanism for certain sterically unencumbered substrate–catalyst combinations.^33,37,38,45,46^ Bulky *t*BuVE, however, is predicted to bind to **HII** only after complete dissociation of the Ru–O bond. Specifically, the barrier for the interchange mechanism via transition state **TS-IC’** is 29.5 kcal/mol (Figure S19), more than 7 kcal/mol higher than that of the dissociative pathway shown in Figure 2 (22.3 kcal/mol, via **TS2-3′**). For the two CAAC catalysts, the question of whether *t*BuVE binding occurs via an interchange or a dissociative mechanism is unimportant for the kinetics of initiation, as the subsequent cycloaddition step is rate-limiting. Any contribution from an interchange pathway for these catalysts would merely lower the olefin-binding barriers relative to those depicted in Figure 2.

Notwithstanding the widespread presumption that initiation rates are limited by the initial Ru–O^*i*^Pr bond dissociation for Hoveyda-class catalysts,^29−32,47^ detailed mechanistic studies (including the present investigation) show that steps subsequent to the formation of **2** are in fact more relevant.^28,37^ Whereas metathesis of vinyl ethers is a terminal, irreversible manifold, an experimental and computational study likewise pointed to cycloaddition as rate-limiting in initiation of **HII** with 1-alkenes such as propene and 1-hexene.^37^ Indeed, the rates of initiation or productive metathesis may be limited by steps even later in the reaction sequence: for example, cycloreversion or product dissociation (in particular for sterically more demanding substrates or catalysts).^28,48−51^ Further strengthening the relevance of the present study is the fact that cycloaddition is a bond-breaking and bond-forming event central to all metathesis reactions, not merely initiation.

The initiation barriers of Figure 2 are consistent with the experimental order of initiation rates: that is, **HII** > **nG-C1**^**Ph**^ > **HC1**^**Ph**^ (Figure 1 and Table 1). This further supports identification of **TS2-3′** and **TS3-4** as the rate-limiting transition states for initiation of **HII** and the CAAC complexes, respectively. Whereas the barriers **1** → **TS3-4** are very similar for all CAAC rotamers, minor energy differences are predicted for the rotamers of **1** and **TS1-2** (0.7–1.1 kcal/mol; see solid vs dashed lines).

### Origin of Differences in Initiation Rates

The impacts of the carbene ligand and the nitro substituent on the free-energy profiles of Figure 2 were analyzed by comparing **HII** with its CAAC analogue **HC1**^**Ph**^, and **HC1**^**Ph**^ with its nitro-benzylidene analogue **nG-C1**^**Ph**^.

### Impact of the Carbene Ligand

Although **HII** initiates faster than **HC1**^**Ph**^, initial rupture of the Ru–O*^i^*Pr bond is slower, the barrier via **TS1-2** being 0.5–1.2 kcal/mol higher for **HII** than **HC1**^**Ph**^. Similarly, the 14-electron complex **2** is more stable for **HC1**^**Ph**^ than **HII** (by 0.7–1.3 kcal/mol; see red vs black lines in Figure 2). The greater stability of **TS1-2** and **2** for **HC1**^**Ph**^ is expected from the stronger trans influence/trans effect of the CAAC ligand noted above.^24,26^

Coordination of *t*BuVE to form π-complex **3** or **3′** (in which the alkene is oriented essentially parallel or orthogonal, respectively, to the Ru=C bond) is strongly endergonic, owing to steric repulsion between the carbene ligand and the alkylidene and alkene moieties. Repulsion will be stronger in the NHC system, as judged from the shorter carbene–alkylidene/alkene distances in the π-complex of **HII** vs **HC1**^**Ph**^ (Figure S22), and the preceding transition states **TS2-3** and **TS2-3′**. This may seem counterintuitive, given the slightly greater bulk of **C1**^**Ph**^ (in terms of buried volume) than H_2_IMes.^24,26^ Access to sterically less encumbered conformations for the **C1**^**Ph**^ catalysts is due to the pronounced difference in bulk of the substituents at the quaternary CMePh site, vs the NAr group. This difference enables access to staggered conformations (via rotation about the carbene C–C and C–N bonds) that relieve steric pressure to an extent unattainable for the NHC ligand. Steric repulsion in the CAAC π-complexes is further reduced by elongation of the Ru–alkene bonds, a consequence of the stronger trans influence of **C1**^**Ph**^ relative to H_2_IMes. This elongation explains why the π-complexes for the two classes of carbene are comparably unstable relative to the precatalyst, despite the greater trans influence of **C1**^**Ph**^.

In summary, the instability of the π-complexes and the associated transition states **TS2-3** and **TS2-3′** is largely steric in origin. For **HII**, with its symmetric H_2_IMes ligand, these effects cause *t*BuVE binding via **TS2-3′** to be rate-determining.^52^ The importance of steric factors also implies, however, that olefin binding is unlikely to be rate-limiting for substrates significantly smaller than *t*BuVE.

**Figure 3:**
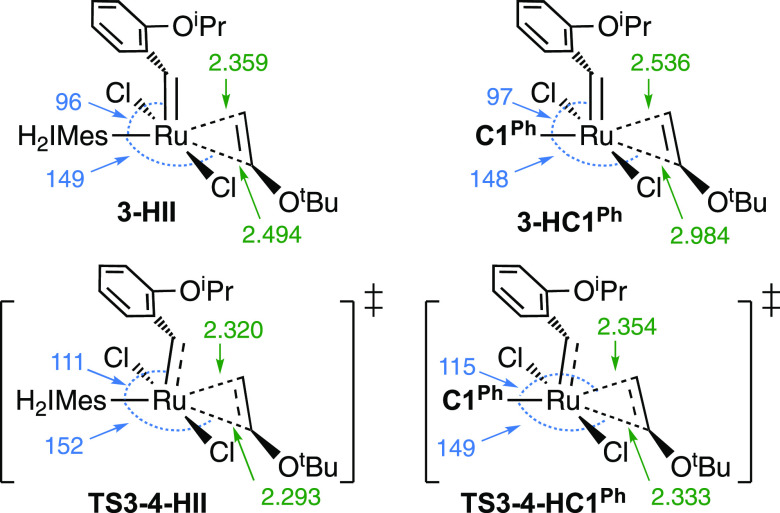
Ru–C_olefin_ bond distances (Å, in green) and C_carbene_–Ru–C_olefin_ bond angles (°, in blue) in the geometry-optimized structures of **3** and **TS3-4** for **HII** and **HC1**^**Ph**^.

Steric factors decline in importance as the alkene and the alkylidene moieties approach each other via **TS3-4** to form metallacyclobutane **4**. The drop in steric pressure is associated with wider bond angles between the carbene ligand and both the alkylidene and the alkene CHO^*t*^Bu moiety in **4**, relative to **3** (Figure 3). As the alkene approaches the ruthenium center along the reaction trajectory from **3** to **TS3-4**, the trans effect of the carbene ligand becomes steadily more important, and the greater trans effect of **C1**^**Ph**^ inhibits cycloaddition. In contrast with the H_2_IMes ligand (which *promotes* cycloaddition by ensuring a remarkably low 1.9 kcal/mol barrier to this step from **3**), the **C1**^**Ph**^ ligand adds more than 5 kcal/mol to this barrier.

### Impact of the Nitro Group: Comparing HC1^Ph^ and nG-C1^Ph^

The nitro substituent lowers the energy of both π-complex **3** and transition state **TS3-4**. Minimal impact is seen on the stability of 14-electron species **2** (derived from **1** by dissociation of the ether donor and ca. 180° rotation of the benzylidene unit, which together comprise a single elementary step). The thermodynamic impact is negligible (0.4 or 0.2 kcal/mol, respectively, depending on whether the N-aryl or the quaternary CMePh is syn to the alkylidene; see Figure 2 and Table S3). This observation was unexpected: weakening of the Ru–ether bond is widely accepted as the basis for the faster initiation of the nitro-Grela catalyst relative to its Hoveyda predecessor.^29−32^

This first elementary step was studied in detail by Solans-Monfort and co-workers, who explored by DFT methods the role of π-delocalization over the Ru=CHC_6_H_4_-2-O^*i*^Pr entity in stabilizing the **HII** precatalyst **1**.^47^ Delocalization increases the π-character in the RuCH–Ar bond, increasing the net barrier to ether dissociation–rotation for **HII**, relative to **nG**. The same effects are seen in the present work: in comparing **HC1**^**Ph**^ and **nG-C1**^**Ph**^, the nitro substituent lowers the barrier to dissociation–rotation by ca. 2 kcal/mol. As noted above and in Figure 2, however, the initiation reactions we examine here are not limited by the initial O^*i*^Pr dissociation–rotation events to yield **2**, but by subsequent steps. A key question is therefore the means by which the nitro group stabilizes **TS3-4**, the transition state that actually limits the rates of initiation of **HC1**^**Ph**^ and **nG-C1**^**Ph**^. Examined below are the factors that underlie the accelerating impact of the nitro group.

**Figure 4:**
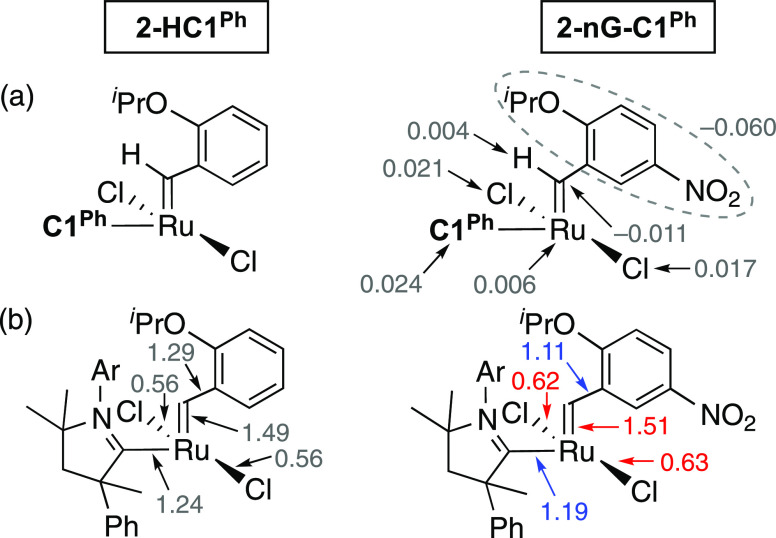
(a) Differences in natural charges, *e*, for selected atoms and fragments in **2-nG-C1**^**Ph**^ compared to **2-HC1**^**Ph**^. (b) Selected natural bond orders (NBOs) illustrate the polarizing effect of the nitro group in the first coordination sphere. NBOs in red for **2-nG-C1**^**Ph**^ indicate increases vs **HC1**^**Ph**^; NBOs in blue indicate decreases. NBOs are obtained by natural resonance theory analysis.^53^ For details, see the SI, Section S2.2.

The polarizing effects of the nitro group are truly long-range. Introducing a nitro substituent causes a buildup of electron density at the aryl group, lowering its overall natural charge by 0.060 *e*, with an additional drop of 0.011 *e* at the alkylidene carbon (Figure 4a). Nearly all of this electron density is supplied by the remote carbene and chloride ligands. Accordingly, the overall natural charge on the **C1**^**Ph**^ ligand increases by 0.024 *e*, while the natural charge on the two chloride ligands increases by 0.021 and 0.017 *e*. The ruthenium atom and the alkylidene hydrogen atom contribute to a relatively minor extent, at 0.006 and 0.004 *e*, respectively. The minuscule increase in charge at the alkylidene hydrogen atom (which is limited to inductive contributions of electron density) suggests that most of the charge polarization induced by the nitro group originates in mesomeric effects.

Natural Resonance Theory (NRT) analysis reveals that many more resonance structures (37 vs 15) are required to describe the electron density of the nitro-Grela complex **2-nG-C1**^**Ph**^ than its Hoveyda analogue **2-HC1**^**Ph**^, consistent with more extensive π-delocalization in the former. Evident from the resonance structures and their weightings (Figures S14–S16) are long-range mesomeric effects that have significant impacts on the bond distances and bond orders in the first coordination sphere around ruthenium. Of particular note, the nitro group causes a significant reduction in the natural bond order of the RuCH–Ar bond (by 0.18; Figure 4b), consistent with the findings of Solans-Monfort and co-workers described above.^47^ Likewise reduced are the Ru–CAAC bond orders, whereas the Ru=CHAr and Ru–Cl bonds are strengthened.

**Figure 5:**
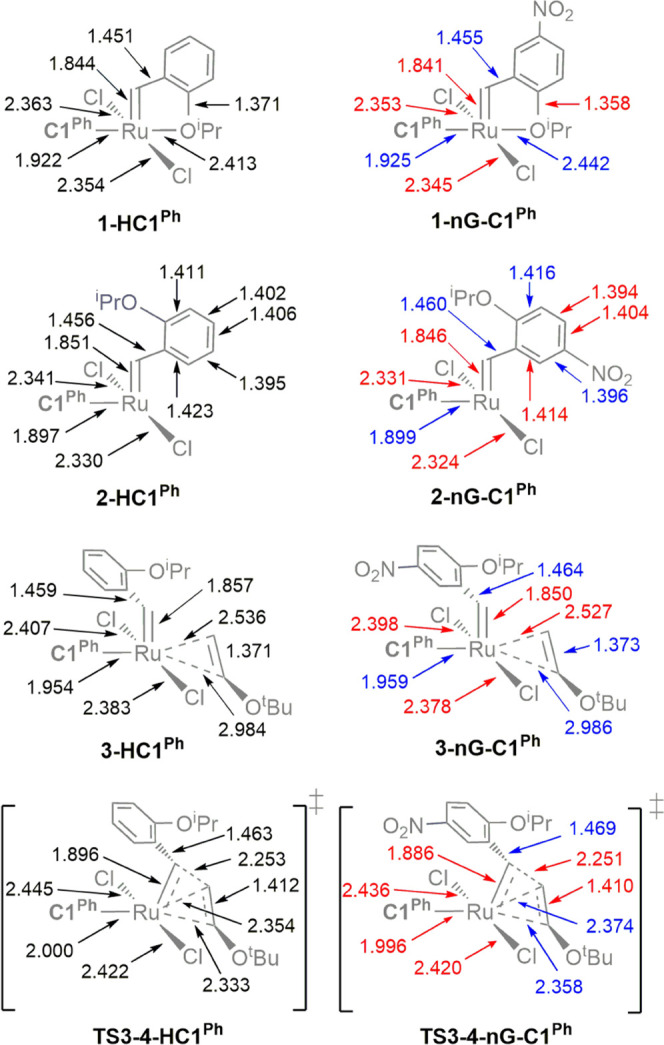
Selected bond distances (Å) in the geometry-optimized structures of **1**–**3** and **TS3-4**. (a) For **HC1**^**Ph**^. (b) For **nG-C1**^**Ph**^, showing contracted (red) or elongated (blue) interatomic distances vs **HC1**^**Ph**^.

These changes in bond order are reflected in the corresponding bond distances (Figure 5). The Ru=CHAr and Ru–Cl distances, in particular, are shorter in **2-nG-C1**^**Ph**^ than **2-HC1**^**Ph**^. An important consequence is increased repulsion within the alkylidene–carbene and Cl–carbene moieties. Steric interactions with the contracted, essentially in-plane Cl_2_Ru=C(alkylidene) fragment, in turn, elongate the out-of-plane Ru–carbene and Ru–alkene bonds. This elongation—a secondary effect of long-range polarization by the nitro group—reduces the mutual trans influence of **C1**^**Ph**^ and the trans-disposed alkene ligand, facilitating olefin binding and cycloaddition via **TS3-4**. The energy of the latter transition state is particularly sensitive to changes in the trans effect of **C1**^**Ph**^, given the near-trans disposition of the carbene and the Ru–CH(O^*t*^Bu) bond (**C1**^**Ph**^–Ru–C_α_ > 170°), and the considerable shortening of the latter bond in this transition state, vs the π-complex. The long-distance mesomeric effects of the nitro group culminate in a remarkable 2 kcal/mol transition-state stabilization, and elongation of the Ru–CH(O^*t*^Bu) bond by 2–3 pm compared to **HC1**^**Ph**^ (Figure 5).

In sum, the mesomeric effects of the nitro substituent reduce the barrier to initiation by modulating key bond orders and distances, and ultimately limiting the destabilizing trans effect of the CAAC ligand in the rate-determining transition state for initiation. The profound impact of these long-range mesomeric effects stands in strong contrast with the minor impact of inductive effects established for fluoro and trifluoromethyl substituents.^32^

### Solvent Effects: Chloroform vs Benzene

**Figure 6:**
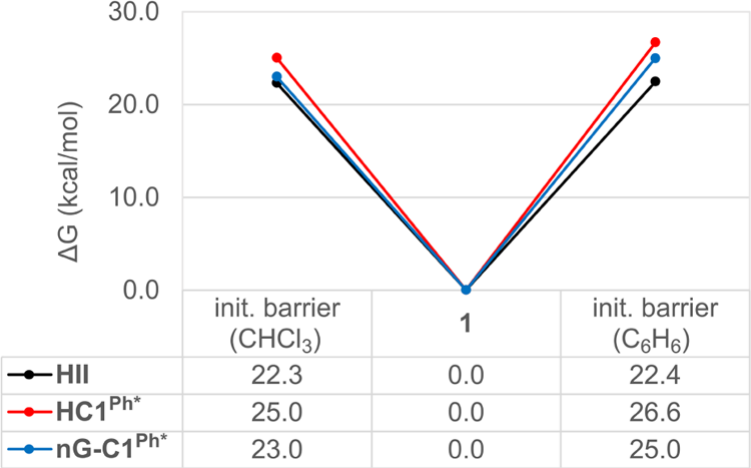
Initiation barriers (i.e., the free energy of **TS2-3′** vs **1** for **HII** and the free energy of **TS3-4** vs **1** for **HC1**^Ph*^ and **nG-C1**^Ph*^) in CHCl_3_ and C_6_H_6_ for the most stable rotamer of the CAAC precatalysts. For a complete list of energies in both solvents, see Table S3.

The observed solvent-dependent trends in initiation rates (Figure 1 and Table 1) are qualitatively reproduced by the initiation barriers estimated for benzene and chloroform solvent using an implicit solvent model (the polarizable continuum model, PCM). The dominant solvent effects are hence macroscopic (Figure 6). The calculations predict that the barrier to initiation for **HII** should be little affected by the choice of solvent, consistent with the experimental data. Intriguingly, the minor influence of solvent seems to originate in the nature of the rate-determining transition state, not the nature of the catalyst. For **HII**, initiation is limited by alkene binding (via **TS2-3′**). However, the energies of the transition states for alkene binding are in general comparatively unaffected by the choice of solvent, even for the CAAC catalysts (Table S3). In contrast, the cycloaddition transition states **TS3-4** are seen to be stabilized by chloroform, relative to benzene. Catalysts for which initiation is limited by cycloaddition (for example, **HC1**^**Ph**^ and **nG-C1**^**Ph**^) are predicted to initiate faster in chloroform than in benzene, exactly as observed experimentally.

## Conclusions

The foregoing establishes a direct comparison of the initiation process for metathesis catalysts of the Grela and Hoveyda classes, in which key NHC and CAAC ligands are present. The CAAC ligand examined is selected for its capacity to limit catalyst decomposition. Its Hoveyda-class derivative **HC1**^**Ph**^ is shown to initiate ca. 100× slower in benzene than the H_2_IMes analogue **HII**, or 40× slower in CDCl_3_. This rate retardation is offset on shifting to the nitro-substituted Grela analogue **nG-C1**^**Ph**^, which initiates approximately 10-fold faster in either solvent.

Slow initiation of the **C1**^**Ph**^ catalysts is traced to the high trans effect/trans influence of the CAAC ligand. The latter electronic property is emerging as key to understanding the major behavioral differences between the CAAC and NHC catalysts. We recently reported its profound impact on decomposition of Ru–CAAC catalysts: here, we find that it is equally relevant to productive metathesis. In short, this simple property has a multitude of impacts just beginning to be recognized. Highlighted herein is its role in destabilizing the transition state for cycloaddition, in which the alkene—itself a high trans influence/trans effect ligand—is tightly bound trans to the carbene. This destabilization is significant (>5 kcal/mol for **HC1**^**Ph**^ vs **HII**). In consequence, [2 + 2] cycloaddition is rate-determining for the CAAC catalysts. For **HII**, in contrast, alkene binding is rate-determining, owing to the rigidity and symmetrical distribution of steric bulk of the H_2_IMes ligand, which impedes the approach of bulky substrates.

An important, little-considered role for the nitro substituent on the benzylidene ring emerged from DFT studies of the Grela complex **nG-C1**^**Ph**^. We find that the nitro group accelerates initiation not by weakening the Ru–O^*i*^Pr bond (as widely believed), or by facilitating rotation of the benzylidene unit to form 14-electron complex **2**, but by strengthening the Ru–alkylidene and Ru–Cl bonds via long-range mesomeric effects. The stronger, shorter bonds of the near-planar Cl_2_Ru=C(alkylidene) unit weaken and elongate the out-of-plane Ru–carbene and Ru–alkene bonds: this attenuates mutual trans effects, hence stabilizing the transition state for cycloaddition.

These findings concerning the role of mesomeric acceleration are broadly relevant for olefin metathesis, irrespective of whether the rate-limiting transition state is cycloaddition (as with vinyl ethers), cycloreversion, or even decoordination of the alkene product. In all cases, the nitro group will reduce the trans effect between the carbene ligand and the trans-bound alkene, resulting in faster initiation. Correspondingly broad implications can be recognized for catalyst design and function. As one instance, mesomeric acceleration effected by redesign of the anionic ligands offers opportunities to accelerate not only initiation, but productive metathesis. This and other avenues are now under study in our laboratories.

## Experimental Section

### General Procedures

Reactions were carried out under N_2_ using glovebox or Schlenk techniques. NMR solvents (CDCl_3_ and C_6_D_6_; Cambridge Isotopes) and *tert*-butyl vinyl ether (*t*BuVE, Sigma-Aldrich, 98%) were freeze/pump/thaw degassed until no bubbles appeared on thawing (minimum 3×) and stored under N_2_ in the glovebox; *t*BuVE at −35 °C, and NMR solvents at RT over 4 Å molecular sieves for at least 12 h prior to use. 1,3,5-Trimethoxybenzene (TMB; 98%, TCI), dimethyl terephthalate (DMT; Sigma-Aldrich, 99%), and anthracene (Sigma-Aldrich, 96%) were used as received. The NHC catalyst **HII**^8^ and the CAAC catalyst^17^**HC1**^**Ph**^ were prepared by literature methods; **nG-C1**^**Ph**^ was kindly supplied as a gift by Apeiron Synthesis.

NMR spectra were recorded on an AVANCE III 500 spectrometer at 25 ± 0.1 °C. Integrations were measured relative to TMB, anthracene, or DMT as internal standard, in CDCl_3_ or C_6_D_6_.

### Catalyst Initiation Experiments

Solid catalysts and internal standards were weighed outside the glovebox using an analytical balance for mass accuracy, and then transferred into the glovebox. In a representative procedure, a stock solution was prepared by dissolving **HII** (23.5 mg, 0.0375 mmol) and anthracene (ca. 1.7 mg, 0.010 mmol, 0.25 equiv) in 1.00 mL CDCl_3_ (37.5 mM **HII**). A 400 μL aliquot was transferred to a NMR tube and diluted with 160 μL CDCl_3_. The NMR tube was sealed, wrapped with Parafilm, and transferred to the NMR probe preset to 25 °C to measure the initial integration ratio of catalyst to anthracene. *t*BuVE (40 μL, 0.30 mmol, 20 equiv) was then injected via gas-tight syringe to give a final volume of 600 μL ([Ru] = 25 mM). The septum was immediately covered with Parafilm, the NMR tube was shaken, and the timer was started. Decreases in the integration of the alkylidene signal against that for internal standard were recorded for at least three half-lives. Representative NMR spectra are shown in Figure S1, rate curves in Figure S2a, and pseudo-first-order plots in Figure S3a. This procedure was repeated with the other catalysts, with the minor modifications indicated below.

**nG-C1**^**Ph**^ (25.7 mg, 0.0375 mmol), with TMB (ca. 1.6 mg, 0.010 mmol, 0.25 equiv). Rate curves and pseudo-first-order plots are shown in Figures S4a and S5a, respectively.

**HC1**^**Ph**^ (12.0 mg, 0.0188 mmol), with TMB (ca. 2 mg, 0.009 mmol, 0.5 equiv) in 500 μL CDCl_3_. A 400 μL aliquot diluted with 121 μL CDCl_3_ was injected with tBuVE (79 μL, 0.60 mmol, 40 equiv). This slow reaction was monitored using the program “kinetic_t_norga.” Rate curves and pseudo-first-order plots are shown in Figures S6a and S7a, respectively.

Experiments in C_6_D_6_ were carried out as above, with **HII** (23.5 mg, 0.0375 mmol) and DMT (ca. 1.6 mg, 0.008 mmol, 0.21 equiv); **nG-C1**^**Ph**^ (25.7 mg, 0.0375 mmol) and DMT (ca. 0.8 mg, 0.004 mmol, 0.11 equiv); **HC1**^**Ph**^ (12.0 mg, 0.0188 mmol) and TMB (ca. 0.3 mg, 0.002 mmol, 0.1 equiv). The corresponding rate curves are shown in Figures S2b, S4b, and S6b, with pseudo-first-order plots in Figures S3b, S5b, and S7b.

## Computational Methods

### Model Building

Initial structure building, conformational searches, and preliminary strain relaxations of molecular models were performed using the implementations of the Merck force field (MMFF94)^54^ and PM6^55^ (a semiempirical method) in Spartan18.^56^ Most of these calculations were performed in conjunction with manually set geometrical constraints in the first coordination sphere of ruthenium, to preserve geometrical features inaccurately described by empirical and semiempirical methods. All density functional theory (DFT) calculations were performed using the B.01^57^ and C.01^58^ versions of the Gaussian 16 suite of programs.

### Geometry Optimization

Geometry optimization was performed using the Gaussian 16 implementation of the generalized-gradient approximation (GGA) functional of Perdew, Burke, and Ernzerhof (PBE),^59,60^ i.e., a functional that does not explicitly account for dispersion interactions. To gauge the effect of the latter, three free-energy differences, Δ*G*1 = *G*(**3**) – *G*(**1**), ΔG2 = *G*(**TS3-4**) – *G*(**1**), and Δ*G*3 = *G*(**4**) – *G*(**1**) for catalyst **HII**, were calculated using the dispersion-including PBE-D3M(BJ) functional (described below) for geometry optimization, in addition to the PBE functional alone. The computational protocol to arrive at free energies (as described in the following) was otherwise identical. The Δ*G*s predicted by the modified (18.4, 20.4, and 11.8 kcal/mol) and standard (18.1, 20.0, and 11.6 kcal/mol) protocol are very similar, suggesting that dispersion corrections are not vital for geometry optimization.

All atoms except ruthenium were described by Dunning’s correlation-consistent valence double-ζ plus polarization basis sets (cc-pVDZ),^61,62^ as retrieved from the EMSL basis set exchange database.^63,64^ Ruthenium was described by the Stuttgart 28-electron relativistic effective core potential (ECP28MDF retrieved from the Stuttgart/Cologne group website),^65^ in combination with the correlation-consistent valence double-ζ plus polarization basis set (cc-pVDZ-PP)^65^ retrieved from the EMSL basis set exchange database.^63,64^

The Gaussian 16 “ultrafine” grid was explicitly specified for numerical integration (keyword int = ultrafine), which implies that this grid was used also for the analytical Hessian calculations. Geometries were optimized using tight convergence criteria (max. force 1.5 × 10^–5^ a.u., RMS force 1.0 × 10^–5^ a.u., max. displacement 6.0 × 10^–5^ a.u., RMS displacement 4.0 × 10^–5^ a.u.), without symmetry constraints, using the following convergence criteria for the self-consistent field (SCF) optimization procedure: RMS change in density matrix <1.0 × 10^–9^, max. change in density matrix <1.0 × 10^–7^.

Electrostatic and nonelectrostatic solvation effects in chloroform were taken into account by using the polarizable continuum model (PCM) in combination with the “Dis,” “Rep,” and “Cav” keywords and the built-in program values (dielectric constant, number density, etc.)^66−69^ The solute cavity was constructed using the united atom topological model with atomic radii optimized for Hartree–Fock (termed “UAHF”).^69−72^ All stationary points were characterized by the eigenvalues of the analytically calculated Hessian matrix, confirming the absence (for minima) or presence of a single negative eigenvalue (for transition states). The translational, rotational, and vibrational components of the thermal corrections to enthalpies and Gibbs free energies were calculated within the ideal-gas, rigid-rotor, and harmonic oscillator (IGRRHO) approximations considering a temperature of 298.15 K. Standard IGRRHO was preferred to approximations (often referred to as quasi-harmonic) involving modified treatments of soft modes due its reported higher accuracy in prediction of entropies of association/dissociation,^73−75^ which here is relevant for the barriers to olefin uptake and release.

Energies (*E*_PBE_^CHCl_3_^) and thermal correction (*G*_PBE_^CHCl_3_ 298.15K^) for all molecular models are reported in Table S3.

### Single-Point Energy Calculations

All single-point energy calculations were performed with the Gaussian 16 implementation of the GGA functional of Perdew, Burke, and Ernzerhof (PBE),^59,60^ including Grimme’s D3 empirical dispersion term^76^ in conjunction with revised Becke–Johnson damping (overall labeled PBE-D3M(BJ) for brevity).^77^ Ruthenium was described by the ECP28MDF relativistic effective core potential^65^ accompanied by a correlation-consistent valence quadruple-ζ plus polarization basis set (ECP28MDF_VQZ),^65^ both obtained from the Stuttgart/Cologne Group website.^78^ Carbon and hydrogen atoms were described by valence quadruple-ζ plus polarization (EMSL: cc-pVQZ)^63,64^ basis sets.^62^ All other atoms were described by valence quadruple-ζ plus polarization augmented with diffuse functions (EMSL: aug-cc-pVQZ).^61,63,64,79^ Electrostatic and nonelectrostatic solvation effects in chloroform and benzene were taken into account using the polarizable continuum model (PCM), in combination with the “Dis,” “Rep,” and “Cav” keywords and the built-in program values (dielectric constant, number density, etc.)^66−69^ The solute cavity was constructed using the united atom topological model with atomic radii optimized for Hartree–Fock (termed “UAHF”).^69−72^ Test calculations involving explicit solvent molecules showed that Ru–solvent complexes are highly unstable relative to the precursor complexes **1** and are unlikely to significantly influence catalyst initiation rates (see SI, Section S2.3.3). This suggests that an implicit, continuum solvent model suffices for the current chemistry and purpose.

Numerical integrations were performed with the “ultrafine” grid of Gaussian 16. The self-consistent field (SCF) density-based convergence criterion was set to 10^–5^ (RMS change in density matrix <1.0 × 10^–5^, max. change in density matrix = 1.0 × 10^–3^). Energy values (*E*_PBE-D3M(BJ)_^CHCl_3_^) and (*E*_PBE-D3M(BJ)_^C_6_H_6_^) for all molecular models are reported in Table S3.

### Calculation of Gibbs Free Energies

Gibbs free energies were calculated at 298.15 K, according to eq 1 (chloroform) and eq 2 (benzene):
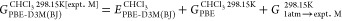
1
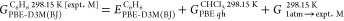
2where *E*_PBE-D3M(BJ)_^CHCl_3_^ and *E*_PBE-D3M(BJ)_^C_6_H_6_^ are the potential energies resulting from the single-point calculation, and include contributions from the implicit solvation model for chloroform and benzene, respectively; *G*_PBE_^CHCl_3_ 298.15K^ is the thermal correction to the Gibbs free energy calculated at the geometry optimization level at 298.15 K; *G*_1atm→expt. M_^298.15K^ is the standard-state correction corresponding to the experimental solution concentrations (catalyst: 0.025 M; *t*BuVE: 0.5 M; 2-isopropoxystyrene: 0.025 M; chloroform: 11.64 M) (but exhibiting infinite-dilution, ideal-gas-like behavior), which at room temperature is equal to −0.29 kcal/mol (= RT·ln(0.6116)) for the catalyst, 1.48 kcal/mol (= RT·ln(12.23)) for *t*BuVE, and 3.35 kcal/mol (= RT·ln(284.8)) for CHCl_3_. Table S3 reports Gibbs free-energy values (*G*_PBE-D3M (BJ)_^CHCl_3_ 298.15K [expt. M]^) and (*G*_PBE-D3M (BJ)_^C_6_H_6_ 298.15K [expt. M]^), and the relative values (Δ*G*_PBE-D3M (BJ)_^CHCl_3_ 298.15K [expt. M]^) and (Δ*G*_PBE-D3M(BJ)_^C_6_H_6_ 298.15K [expt. M]^) calculated with respect to the precatalyst **1**. For **1-nG-C1**^**Ph**^ and **1-HC1**^**Ph**^, two rotamers exist, in which the alkylidene is syn to the N-aryl substituent or to the quaternary CMePh site of the CAAC ligand (Figure S9). The latter, denoted (*), is more stable, and relative energies are calculated with respect to this rotamer.

### Natural Bond Orbital (NBO) and Natural Resonance Theory (NRT) Analyses

NBO^80,81^ and NRT^53^ analyses of **2-HC1**^**Ph**^ and **2-nG-C1**^**Ph**^ were performed with the standalone version (GenNBO) of the NBO 7.0 program,^82^ using the electron density of the single-point energy calculations (via the Gaussian-generated archive file, FILE47).^83^ The latter file was modified and used as the input to GenNBO. To ensure a comparable set of initial resonance structures for **2-HC1**^**Ph**^ and **2-nG-C1**^**Ph**^, reference resonance structures were specified as a list of lone pairs and bonds using the keyword $NRTSTR. Details of the NRT analyses, using both single-reference and multireference initial set of resonance structures, are given in the Supporting Information.

## Supporting Information Available

Spectra, kinetics data, and additional computational data (PDF)
Computed molecular Cartesian coordinates (XYZ)

## Supplementary Material

cs2c03828_si_001.pdf

cs2c03828_si_002.xyz
