# Supplementary material for: Mesomeric Acceleration Counters Slow Initiation of Ruthenium–CAAC Catalysts for Olefin Metathesis (CAAC = Cyclic (Alkyl)(Amino) Carbene)
Source: ACS Catal. 2023 Apr 5;13(8):5315–25. doi: 10.1021/acscatal.2c03828 (PMC10127214; doi:10.1021/acscatal.2c03828)
Supplement: Supplementary file 1 — cs2c03828_si_001.pdf [file cs2c03828_si_001.pdf]

Supporting Information

Mesomeric Acceleration Counters Slow Initiation of Ruthenium–CAAC Catalysts for Olefin Metathesis (CAAC = Cyclic (Alkyl)(Amino) Carbene)

Xinrui Ou,[a] Giovanni Occhipinti,[b] Eliza-Jayne Y. Boisvert,[a] Vidar R. Jensen[b]* and Deryn E. Fogg[a,b]*

[a]Center for Catalysis Research & Innovation, and Department of Chemistry and Biomolecular Sciences, University of Ottawa, Ottawa, Canada K1N 6N5. [b]Department of Chemistry, University of Bergen, Allégaten 41, N-5007 Bergen, Norway

*Corresponding authors: dfogg@uottawa.ca, dfo025@uib.no, vidar.jensen@uib.no

*Table of Content*s

S1. Kinetics Experiments ***S2***

S1.1 Representative NMR Spectra **S2**

S1.2 Kinetics Data **S4**

S2. Computational Study ***S12***

S2.1 Rotamers of CAAC Complexes **S12**

S2.2 Natural Resonance Theory (NRT) Analysis of 2-HC1^{Ph} and 2-nG-C1^{Ph} **S12**

S2.2.1 Single-Reference NRT analysis S12
S2.2.2 Multi-Reference NRT analysis S15
S2.2.3 Input File for NRT Analysis of **2-HC1^{Ph}** S21
S2.2.4 Input File for NRT Analysis of **2-nG-C1^{Ph}** S23

S2.3 Computational Study of the Initiation Pathway **S28**

S2.3.1 Interchange Pathway for **HII** S28
S2.3.1 Complete Pathway for **HII** S29
S2.3.3 Effects of Solvent Coordination on the Initiation Rate S31
S2.3.4 All DFT-Calculated Absolute and Relative Energies S32

S3. References ***S34***

S1. Kinetics Experiments

S1.1 Representative NMR Spectra

(i) Initial spectrum in $CDCl_3$: **HII** + anthracene (internal standard, IS)

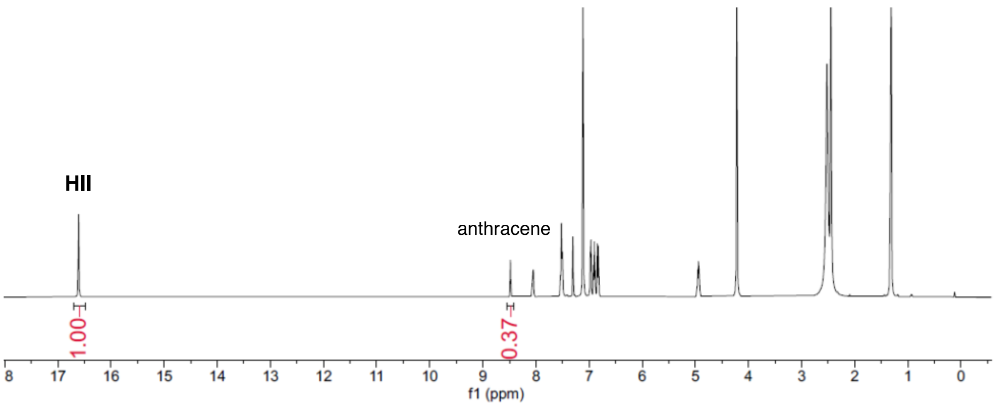

(ii) Control reaction in the absence of tBuVE: 0% decomposition of **HII** after 72 h (3 days). Inset shows starting ratio of **HII**:IS.

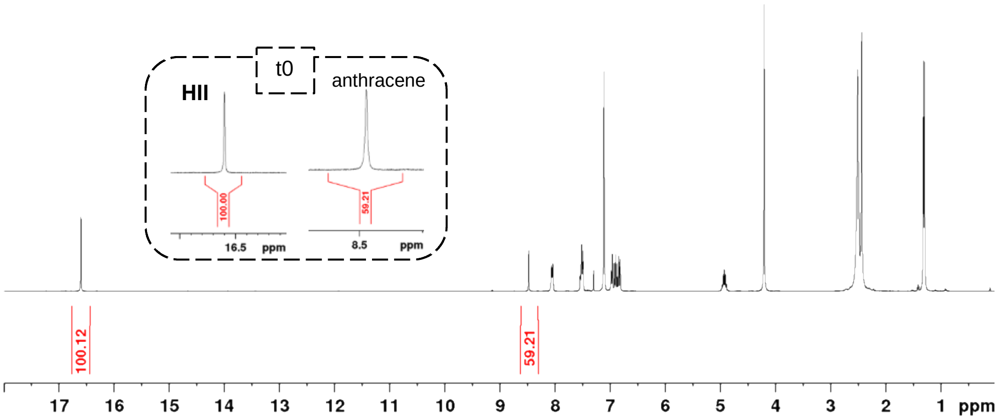

(iii) Sample in (i), recorded 31 min after adding 20 equiv tBuVE

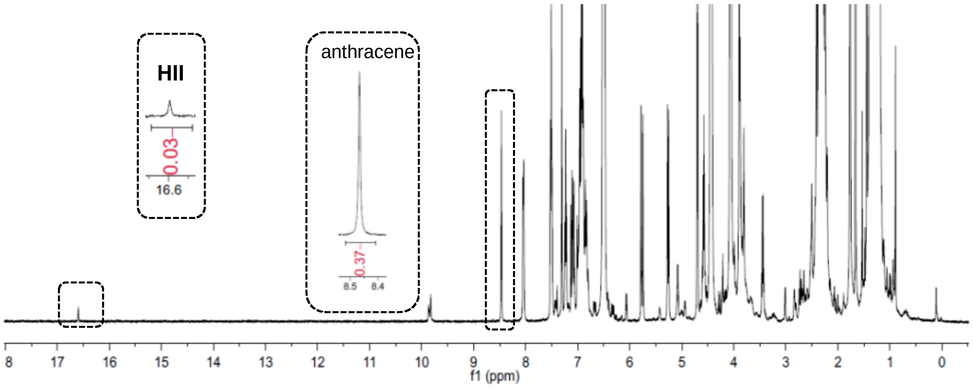

Figure S1a. Representative ^1H NMR spectra for initiation of **HII** in $CDCl_3$ (500 MHz, 25±0.1 °C). (i) Prior to adding tBuVE. (ii) Control experiment in the absence of tBuVE, showing 100% **HII** vs internal standard (IS) after 72 h at RT. Inset shows starting ratio of **HII**:IS. (iii) Sample from (i) after adding tBuVE (20 equiv), recorded 31 min later: 97% consumption of **HII** vs IS.

(i) Initial spectrum in C_6D_6: **HII** + dimethyl terephthalate (DMT; internal standard, IS)

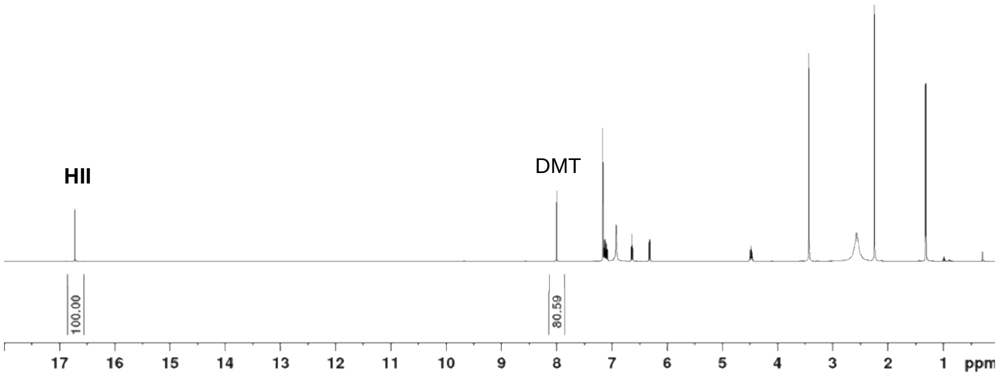

(ii) Control reaction in the absence of tBuVE: 0.4% decomposition of **HII** after 72 h (3 days). Inset shows starting ratio of **HII**:IS.

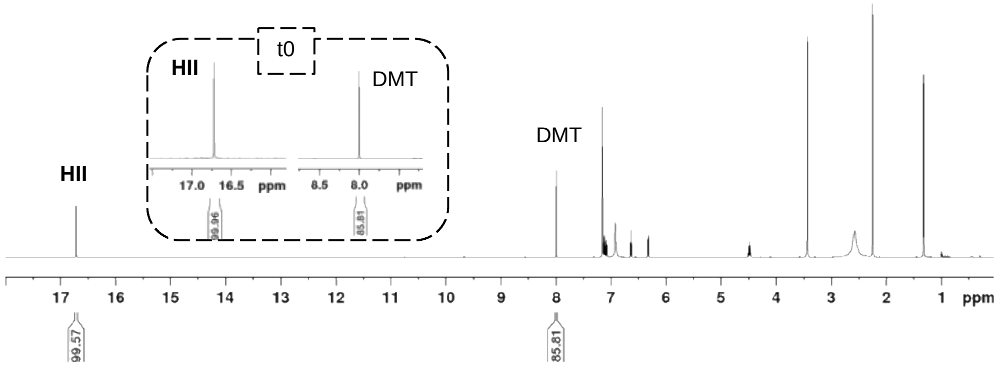

(iii) Sample in (i), recorded 22 min after adding 20 equiv tBuVE

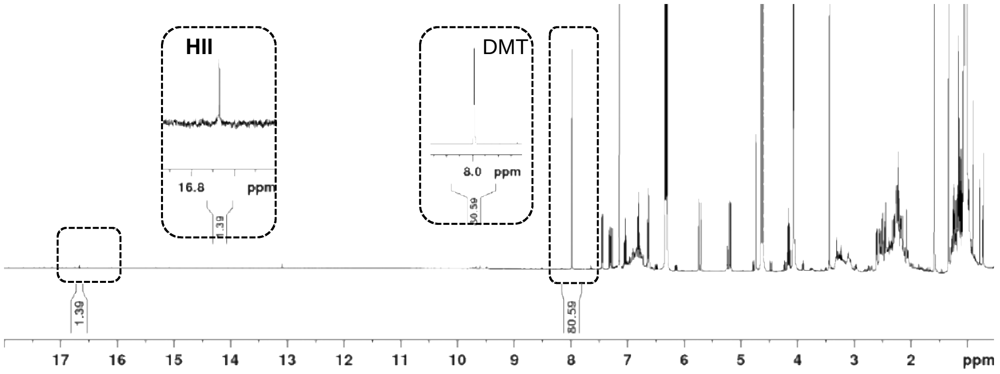

Figure S1b. Representative ^{1}H NMR spectra for initiation of **HII** in C_6D_6 (500 MHz, 25±0.1 °C). (i) Prior to adding tBuVE. (ii) Control experiment in the absence of tBuVE, showing 99.6% **HII** vs internal standard (IS) after 72 h at RT. Inset shows starting ratio of **HII**:IS. (iii) Sample from (i) after adding tBuVE (20 equiv), recorded 22 min later: 99% consumption of **HII** vs IS.

S1.2 Kinetics Data

(a) **HII**, $CDCl_3$

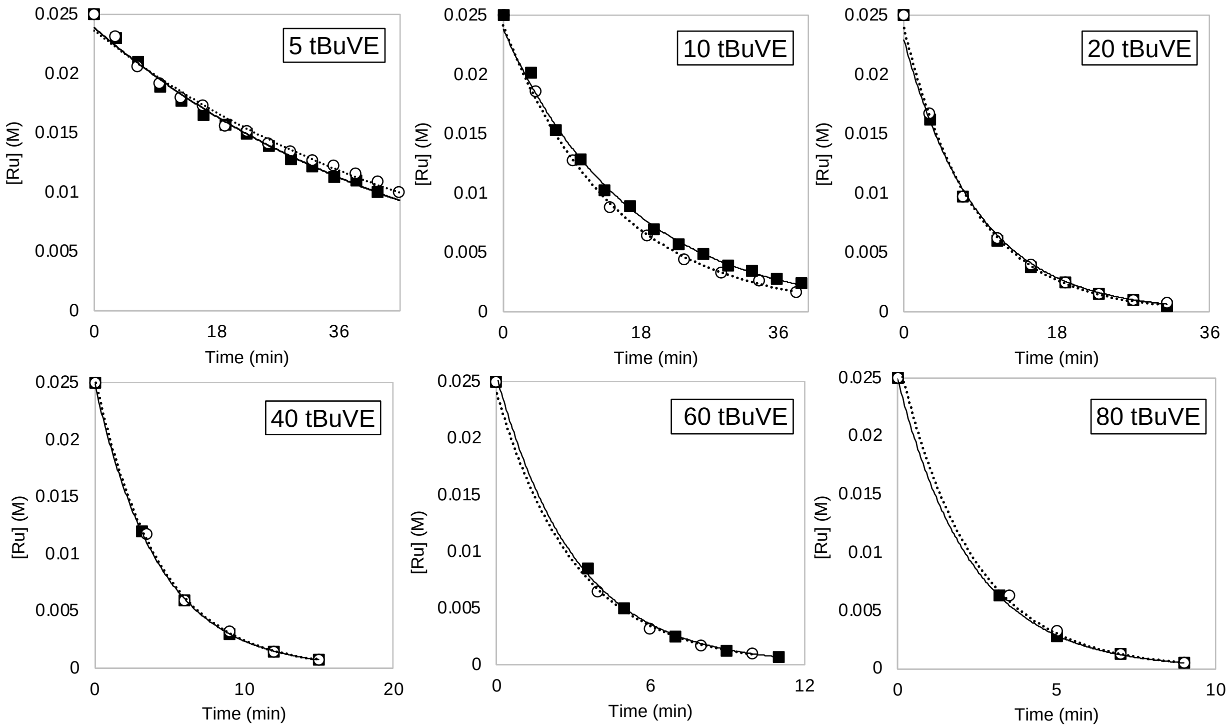

(b) **HII**, C_6D_6

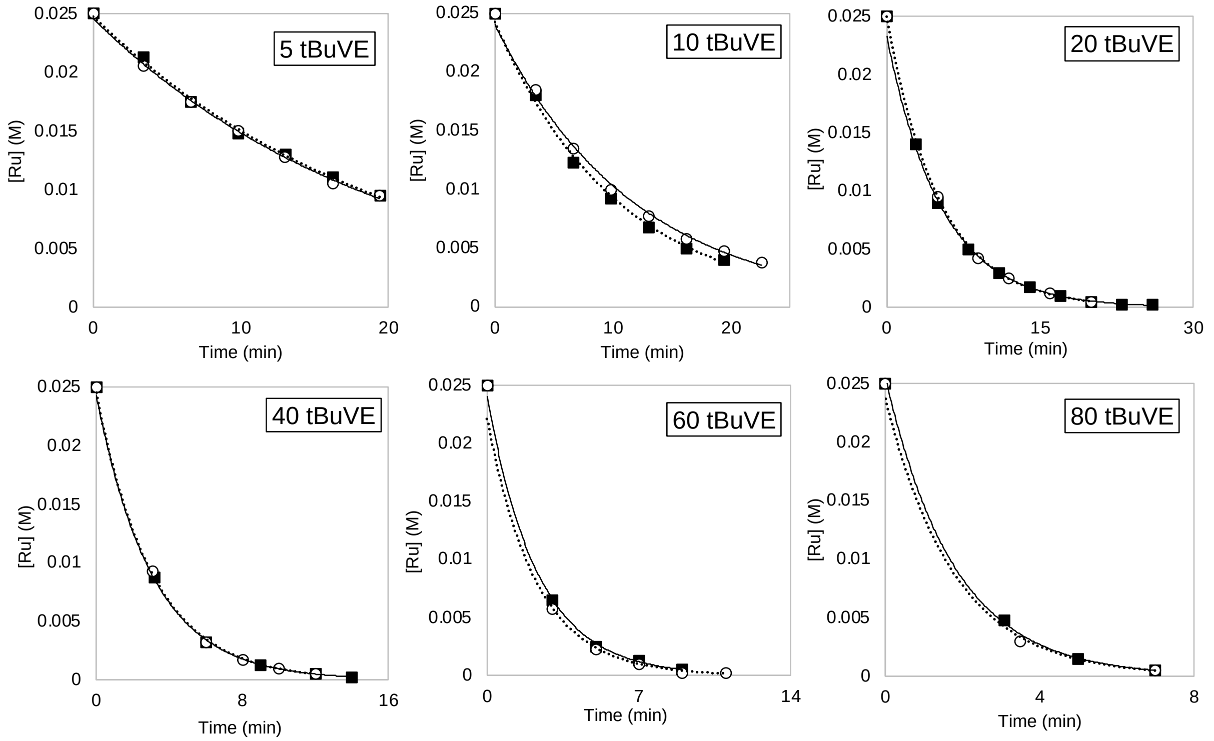

Figure S2. Rate curves for reaction of **HII** with tBuVE at 25±0.1 °C. (a) In $CDCl_3$. (b) In C_6D_6. Dashed lines (○): Trial 1. Solid lines (■): Trial 2.

(a) **HII**, $CDCl_3$

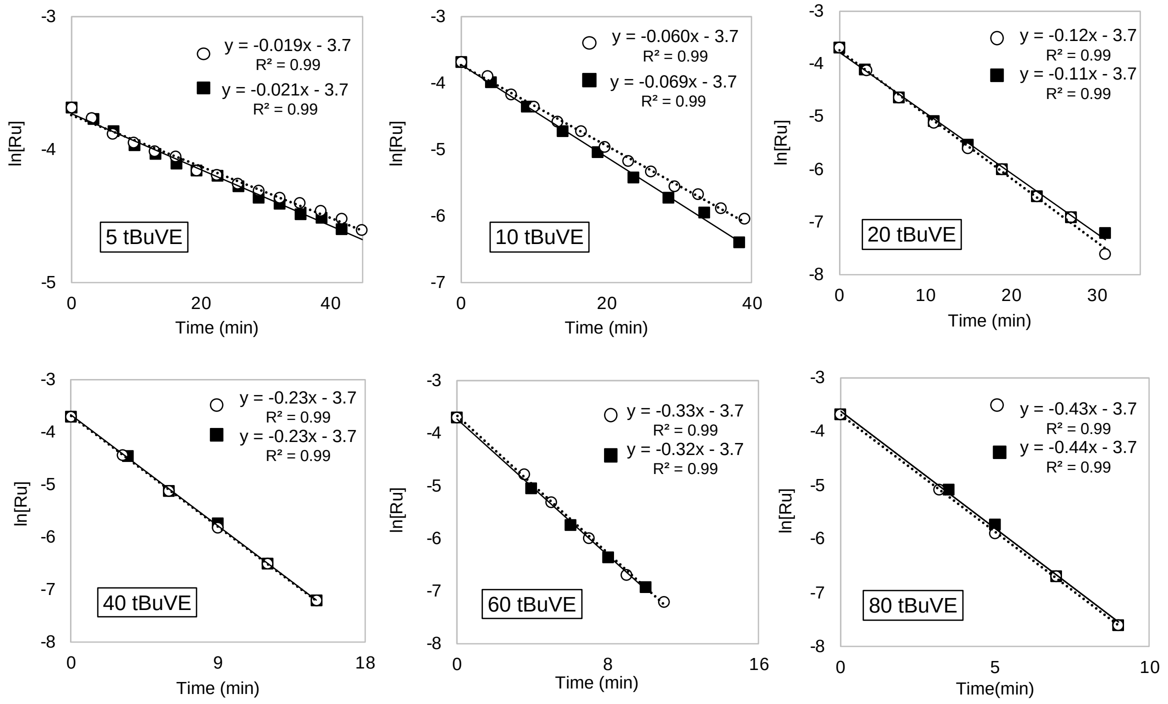

(b) **HII**, C_6D_6

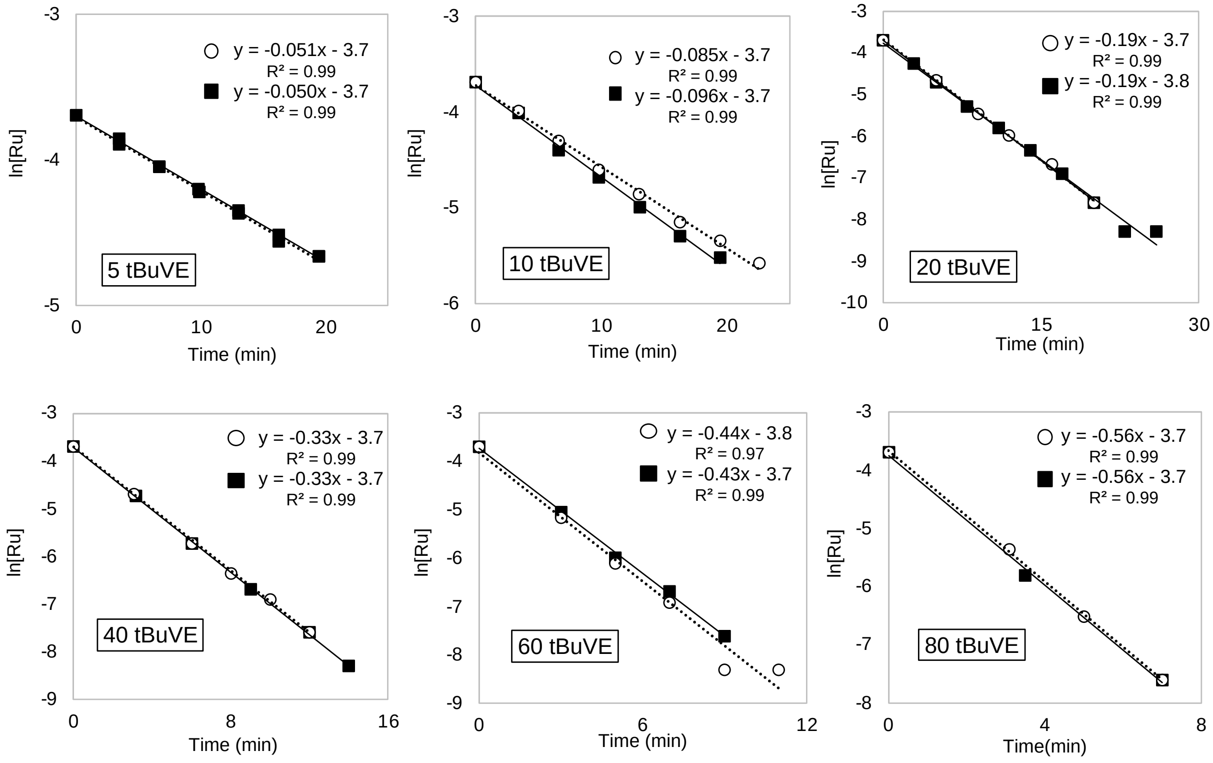

Figure S3. Pseudo-first-order plots for reaction of **HII** with tBuVE at 25±0.1 °C. (a) In $CDCl_3$. (b) In C_6D_6. Dashed lines (○): Trial 1. Solid lines (■): Trial 2.

(a) **nG-C1^{Ph}**, $CDCl_3$

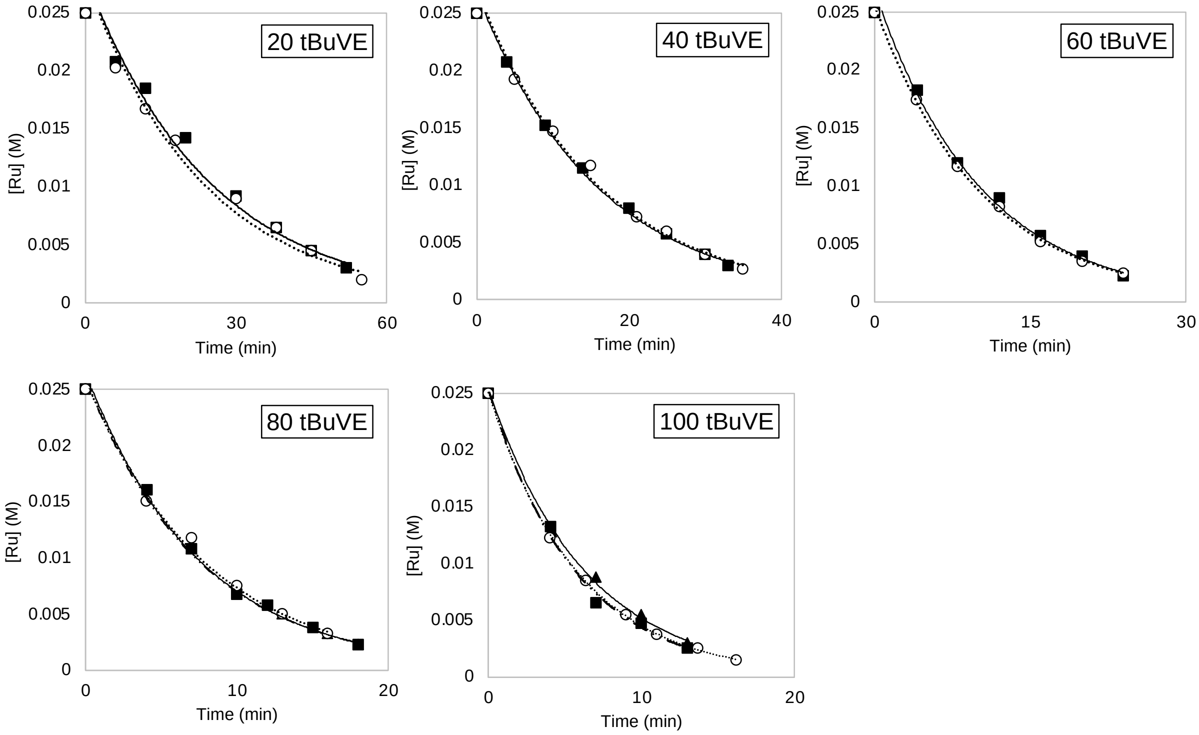

(b) **nG-C1^{Ph}**, C_6D_6

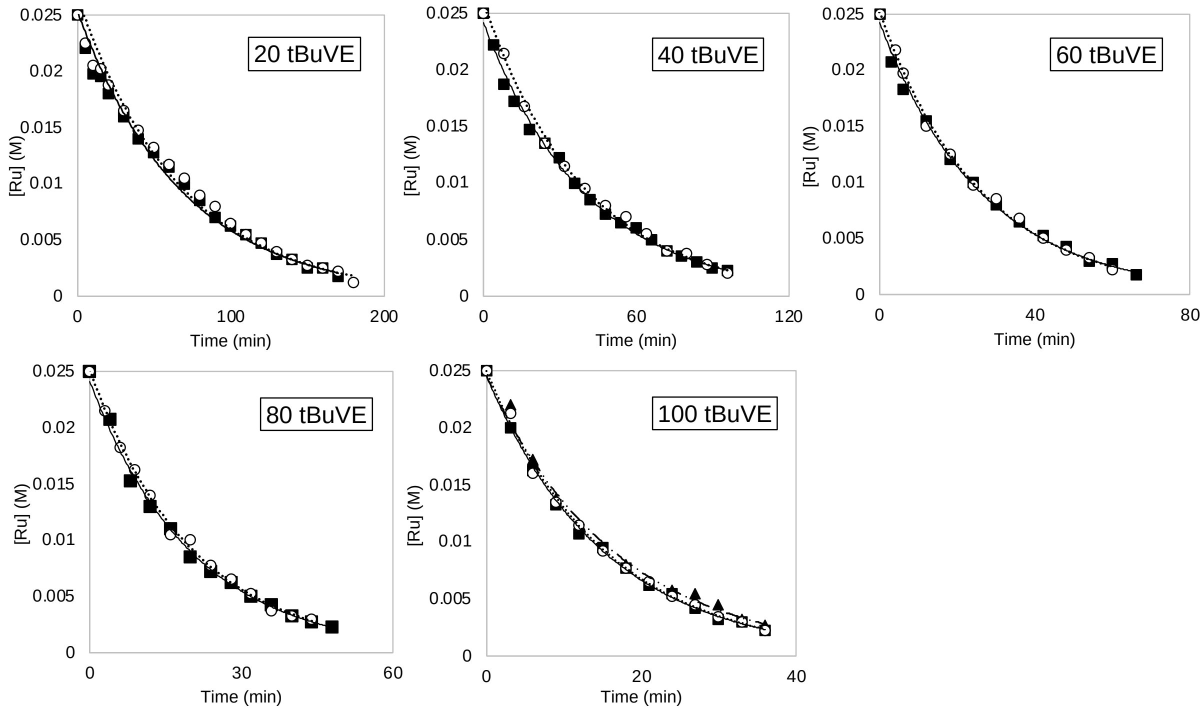

Figure S4. Rate curves for reaction of **nG-C1^{Ph}** with tBuVE at 25±0.1 °C. (a) In $CDCl_3$. (b) In C_6D_6. Dashed lines (○): Trial 1. Solid lines (■): Trial 2. Dashed line (▲): Trial 3 (conducted for 100 tBuVE, as standard deviations between Trials 1-2 were >5%).

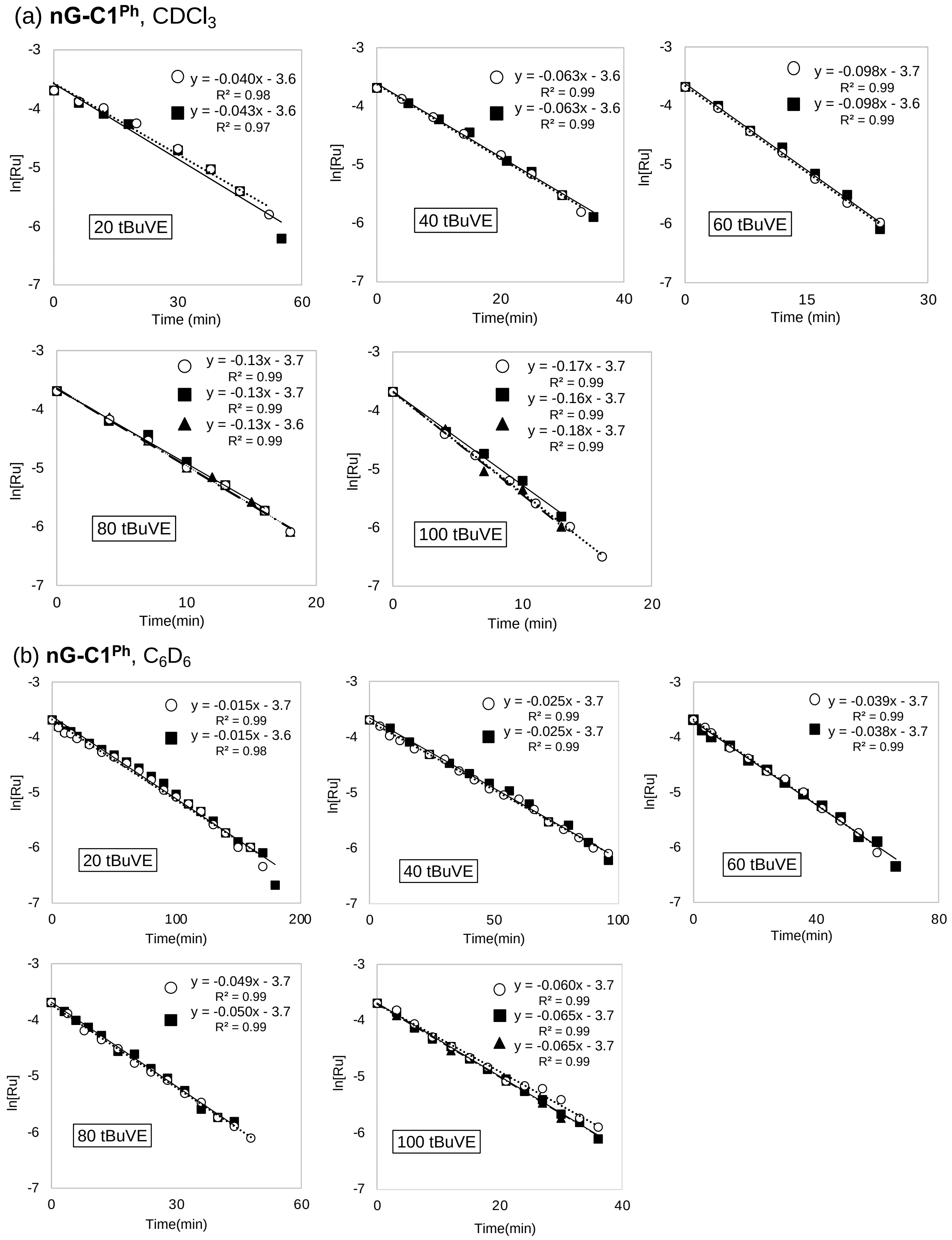

Figure S5. Pseudo-first-order plots for reaction of **nG-C1^{Ph}** with tBuVE at 25±0.1 °C. (a) In $CDCl_3$. (b) In C_6D_6. Dashed lines (○): Trial 1. Solid lines (■): Trial 2. Dashed lines (▲): Trial 3: conducted for 80/100 equiv tBuVE, as standard deviations between Trials 1-2 were >5%.

(a) **HC1**$^{\mathbf{Ph}}$, $CDCl_3$

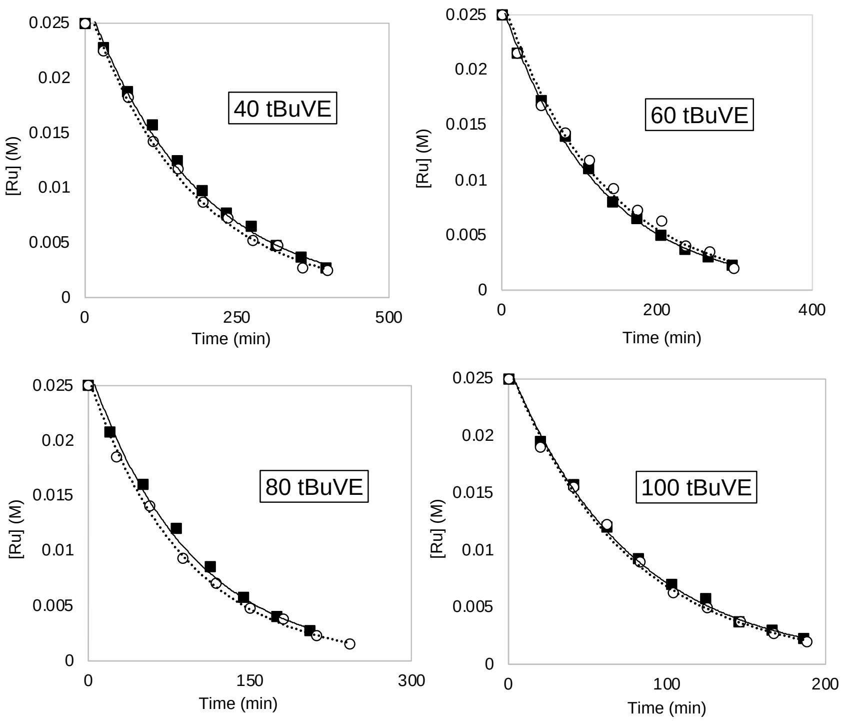

(b) **HC1**$^{\mathbf{Ph}}$, C_6D_6

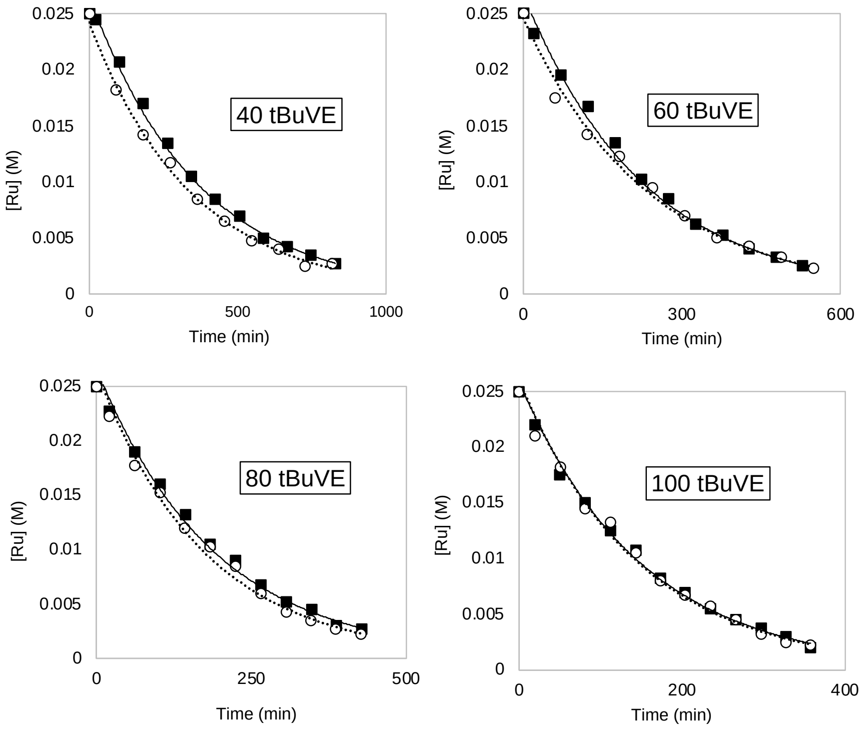

Figure S6. Rate curves for reaction of **HC1**$^{\mathbf{Ph}}$ with tBuVE at 25±0.1 °C. (a) In $CDCl_3$. (b) In C_6D_6. Dashed lines (○): Trial 1. Solid lines (■): Trial 2.

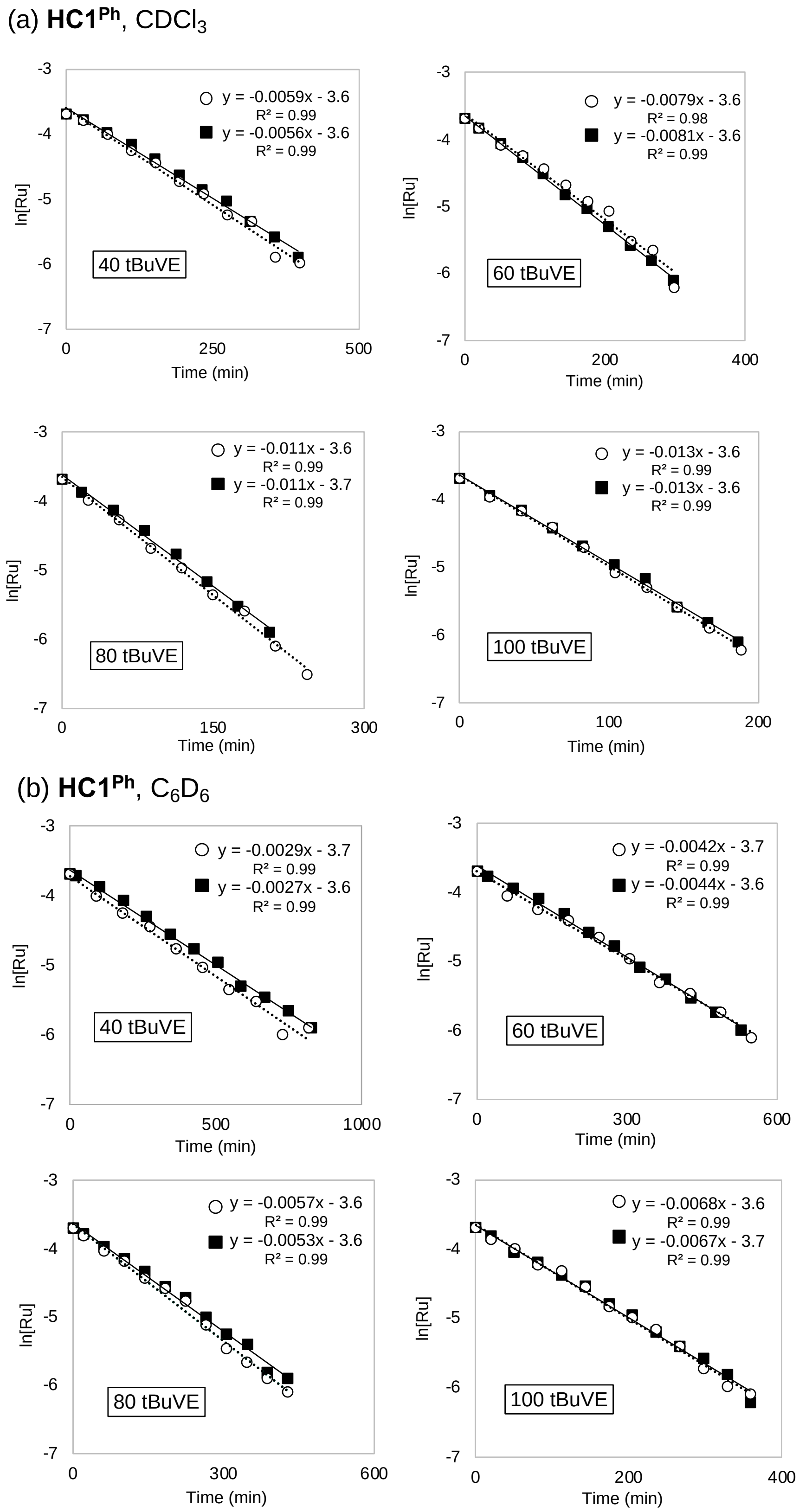

Figure S7. Pseudo-first-order plots for reaction of **HC1**$^{\mathbf{Ph}}$ with tBuVE at 25±0.1 °C. (a) In $CDCl_3$. (b) In C_6D_6. Dashed lines (○): Trial 1. Solid lines (■): Trial 2.

Table S1. Summary of pseudo-first-order rate constants ($k_{obs} = k_1$[tBuVE]) for **HII**, **$HC1^{Ph}$** and **nG-$C1^{Ph}$**

Catalyst	Solvent	[tBuVE] (M)	k_{obs} (min^{-1}) Trial 1	k_{obs} (min^{-1}) Trial 2	k_{obs} (min^{-1}) Trial 3[a]	Average (min^{-1})
**HII**	$CDCl_3$	0.125	0.0192	0.0209	–	0.0200
**HII**	$CDCl_3$	0.25	0.0604	0.0694	–	0.0649
**HII**	$CDCl_3$	0.51	0.1215	0.1149	–	0.1182
**HII**[b]	$CDCl_3$	0.51	0.1213	–	–	
**HII**	$CDCl_3$	1.00	0.2344	0.2340	–	0.2342
**HII**	$CDCl_3$	1.49	0.3263	0.3241	–	0.3252
**HII**	$CDCl_3$	2.00	0.4328	0.4363	–	0.4346
**HII**	C_6D_6	0.125	0.0505	0.0504	–	0.0504
**HII**	C_6D_6	0.25	0.0847	0.0958	–	0.0902
**HII**	C_6D_6	0.51	0.1926	0.1863	–	0.1895
**HII**	C_6D_6	1.00	0.3272	0.3253	–	0.3263
**HII**	C_6D_6	1.49	0.4449	0.4315	–	0.4382
**HII**	C_6D_6	2.00	0.5589	0.5613	–	0.5601
**$HC1^{Ph}$**	$CDCl_3$	1.00	0.0059	0.0056	–	0.0058
**$HC1^{Ph}$**	$CDCl_3$	1.49	0.0081	0.0079	–	0.0080
**$HC1^{Ph}$**	$CDCl_3$	2.00	0.0113	0.0107	–	0.0110
**$HC1^{Ph}$**	$CDCl_3$	2.50	0.0130	0.0134	–	0.0132
**$HC1^{Ph}$**	C_6D_6	1.00	0.0029	0.0027	–	0.0028
**$HC1^{Ph}$**	C_6D_6	1.49	0.0044	0.0042	–	0.0043
**$HC1^{Ph}$**	C_6D_6	2.00	0.0057	0.0053	–	0.0055
**$HC1^{Ph}$**	C_6D_6	2.50	0.0068	0.0067	–	0.0068
**nG-$C1^{Ph}$**	$CDCl_3$	0.51	0.0404	0.0428	–	0.0416
**nG-$C1^{Ph}$**	$CDCl_3$	1.00	0.0629	0.0634	–	0.0632
**nG-$C1^{Ph}$**	$CDCl_3$	1.49	0.0976	0.0983	–	0.0980
**nG-$C1^{Ph}$**	$CDCl_3$	2.00	0.1266	0.1328	0.1316	0.1303
**nG-$C1^{Ph}$**	$CDCl_3$	2.50	0.1716	0.1593	0.1761	0.1690
**nG-$C1^{Ph}$**	C_6D_6	0.51	0.0146	0.0149	–	0.0148
**nG-$C1^{Ph}$**	C_6D_6	1.00	0.0253	0.0247	–	0.0250
**nG-$C1^{Ph}$**	C_6D_6	1.49	0.0386	0.0377	–	0.0382
**nG-$C1^{Ph}$**	C_6D_6	2.00	0.0494	0.0498	–	0.0496
**nG-$C1^{Ph}$**	C_6D_6	2.50	0.0600	0.0651	0.0652	0.0634

[a]A third trial was conducted in some experiments at high tBuVE, if standard deviations between Trials 1-2 were >5%.
[b]At the request of a reviewer, DMT was used as an alternative internal standard in place of anthracene, to confirm that the nature and potential binding ability of the internal standard has no impact.

(a) In $CDCl_3$

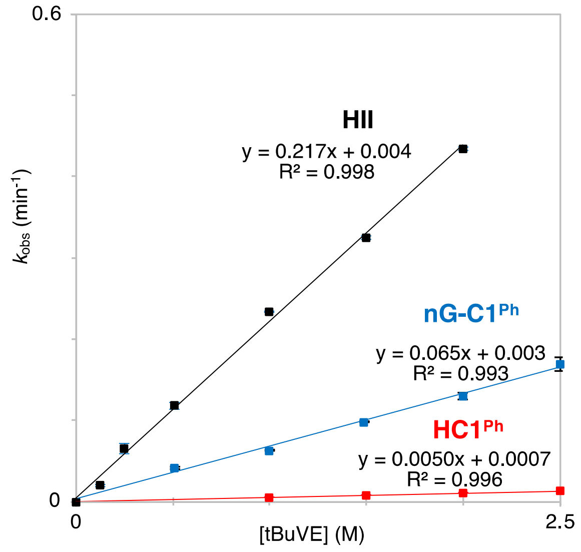

(b) In C_6D_6

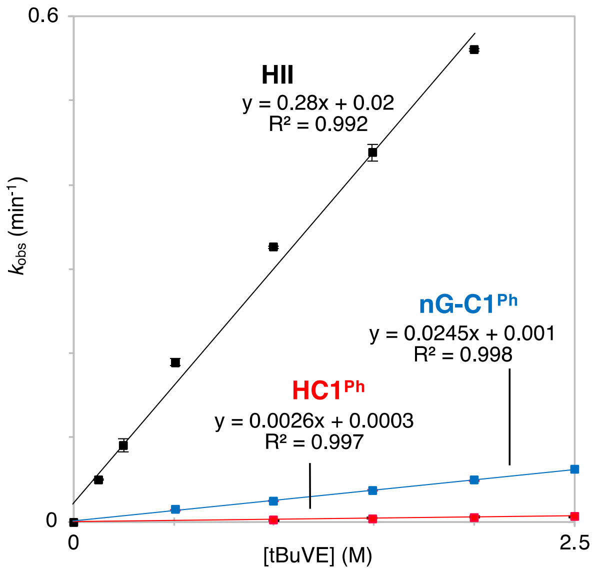

Catalyst	Solvent	Slope	y-Intercept
**HII**	$CDCl_3$	0.217±0.005	0.004±0.005
**HC1^{Ph}**	$CDCl_3$	0.0050±0.0002	0.0007±0.0004
**nG-C1^{Ph}**	$CDCl_3$	0.065±0.003	0.003±0.005
**HII**	C_6D_6	0.28±0.01	0.02±0.01
**HC1^{Ph}**	C_6D_6	0.0026±0.0002	0.0003±0.0002
**nG-C1^{Ph}**	C_6D_6	0.0245±0.0006	0.001±0.001

Figure S8. (Enlarged version of Fig. 1 in the main text, with tabulated values). Second-order plots for reactions of **HII** (black), **nG-C1^{Ph}** (blue), and **HC1^{Ph}** (red) with tBuVE at 25±0.1 °C. (a) Experiments in $CDCl_3$. (b) Experiments In C_6D_6.

S2. Computational Study

S2.1 Rotamers of CAAC Complexes

Within the **C1**^{Ph} species, (*) denotes the rotamer in which the alkylidene is syn to CMePh; that in which it is syn to N-Ar bears no asterisk (Figure S9).

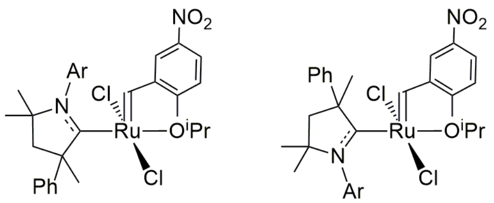

1-nG-C1^{Ph} **1-nG-C1**^{Ph*}

Figure S9. Rotamers of complexes containing the **C1**^{Ph} ligand, illustrated for the nitro-Grela derivative **1-nG-C1**^{Ph}.

S2.2 Natural Resonance Theory (NRT) Analysis of 2-HC1^{Ph} and 2-nG-C1^{Ph}

S2.2.1 Single-Reference NRT analysis

The Lewis structures found by the NBO program[1] for **2-HC1**^{Ph} (Figure S10a) and **2-nG-C1**^{Ph} (Figure S10b) were used as initial input structures for their respective single-reference Natural Resonance Theory (NRT)[2] analyses.

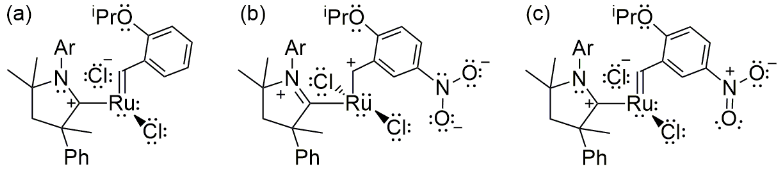

2-HC1^{Ph} (97.8% Lewis) **2-nG-C1**^{Ph} (97.6% Lewis) **2-nG-C1**^{Ph} (97.7% Lewis)

Figure S10. (a) The best Lewis structure found by NBO for **2-HC1**^{Ph}, subsequently used in single-reference NRT analysis. (b) The best Lewis structure found by NBO for **2-nG-C1**^{Ph}. (c) Manually designed Lewis structure for single-reference NRT analysis of **2-nG-C1**^{Ph}.

The Lewis structure in Figure S10a accounts for 97.8% of the total electron density of **2-HC1**^{Ph}. It features an anionic chloride ligand, two lone pairs on the ruthenium atom, and a Ru–alkylidene double bond. In contrast, the structure in Figure S10b accounts for less (by 0.2 percentage points) of the total electron density of **2-nG-C1**^{Ph} and differs significantly from that of Figure S10a. This structure has two negative charges on the nitro group (and thus also on the 4-nitro-isopropoxyphenyl moiety), two Cl atoms covalently bonded to ruthenium, and a Ru–alkylidene single bond. However, a Ru–alkylidene single bond is inconsistent with the relatively short Ru–$C_{alkylidene}$ bond distance (1.846 Å) in the DFT-optimized geometry of **2-nG-C1**^{Ph}, which is even shorter than that of **2-HC1**^{Ph} (1.851 Å).

Moreover, most of the 27 resonance structures generated in the initial single-reference NRT analysis of **2-nG-C1**^{Ph} based on the Lewis structure of Figure S10b feature the same non-physical

negative charges on the nitro group and a Ru–alkylidene single bond. This manifold of secondary structures generated from the Figure S10b reference is essentially disconnected from the manifold generated for **2-HC1^{Ph}** based on the reference in Figure S10a.

To achieve overlapping manifolds (i.e., manifolds that include resonance structures of the same kind), and a fairer comparison of **2-HC1^{Ph}** and **2-nG-C1^{Ph}**, a new reference structure was manually designed for **2-nG-C1^{Ph}**, by replacing the hydrogen ion (H^+) trans to the isopropoxy group in Figure S10a with a classical Lewis representation of a ligated nitronium ion $[NO_2]^+$. The resulting Lewis structure (Figure S10c) accounts for 97.7% of the total electron density, i.e. 0.1% more than that of Figure S10b. The structure of Figure S10c is thus a better representation of **2-nG-C1^{Ph}**, and a better starting point for NRT analysis, than the structure resulting from the NBO search in Figure S10b.

Figures S11 and S12 display the resonance structures and their weights resulting from single-reference NRT analyses of **2-HC1^{Ph}** (Figure S10a) and **2-nG-C1^{Ph}** (Figure S10c), respectively.

No Lewis structures such as that of Figure S10b (with two negative charges on the nitro group, a Ru–alkylidene single bond, and two covalently-bonded Cl atoms) were among the resonance structures generated in the single-reference NRT analysis of **2-nG-C1^{Ph}** (Figure S12). The NRT search appears to be anchored by the reference, and, in the present case, a single-reference NRT analysis is likely to miss important features of the electronic structure of **2-nG-C1^{Ph}**, such as those found by the NBO search and expressed in Figure S10b.

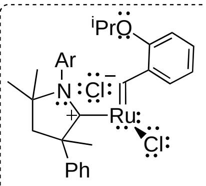

Reference structure

No contribution to Ru-alkylidene bond polarization (weight = 66.4%)

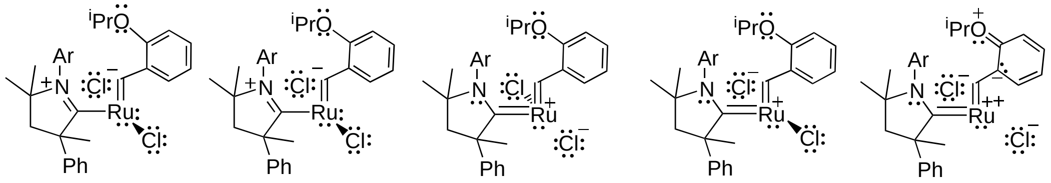

NRT1 (25.60%) NRT4 (10.18%) NRT2 (20.83%) NRT6 (6.20%) NRT7 (3.59%)

Polarization of the Ru-alkylidene bond toward Ru (weight = 29.83%)

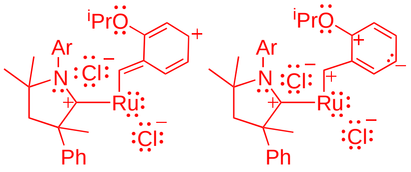

NRT3 (19.87%) NRT5 (9.96%)

Polarization of the Ru-alkylidene bond toward C (weight = 3.09%)

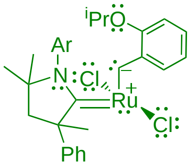

NRT8 (3.09%)

Figure S11. Resonance structures and weights obtained in the single-reference NRT analysis of **2-HC1^{Ph}**, with the minimized NRT objective function (i.e., the root-mean-square deviation of the NRT one-electron reduced density matrix from the original DFT one-electron reduced density matrix, $\Delta(\{w\}) = 0.04120183$). Resonance forms of the substituents Ar (2,6-diethylphenyl), Ph (phenyl), and iPr (isopropyl) are omitted for clarity.

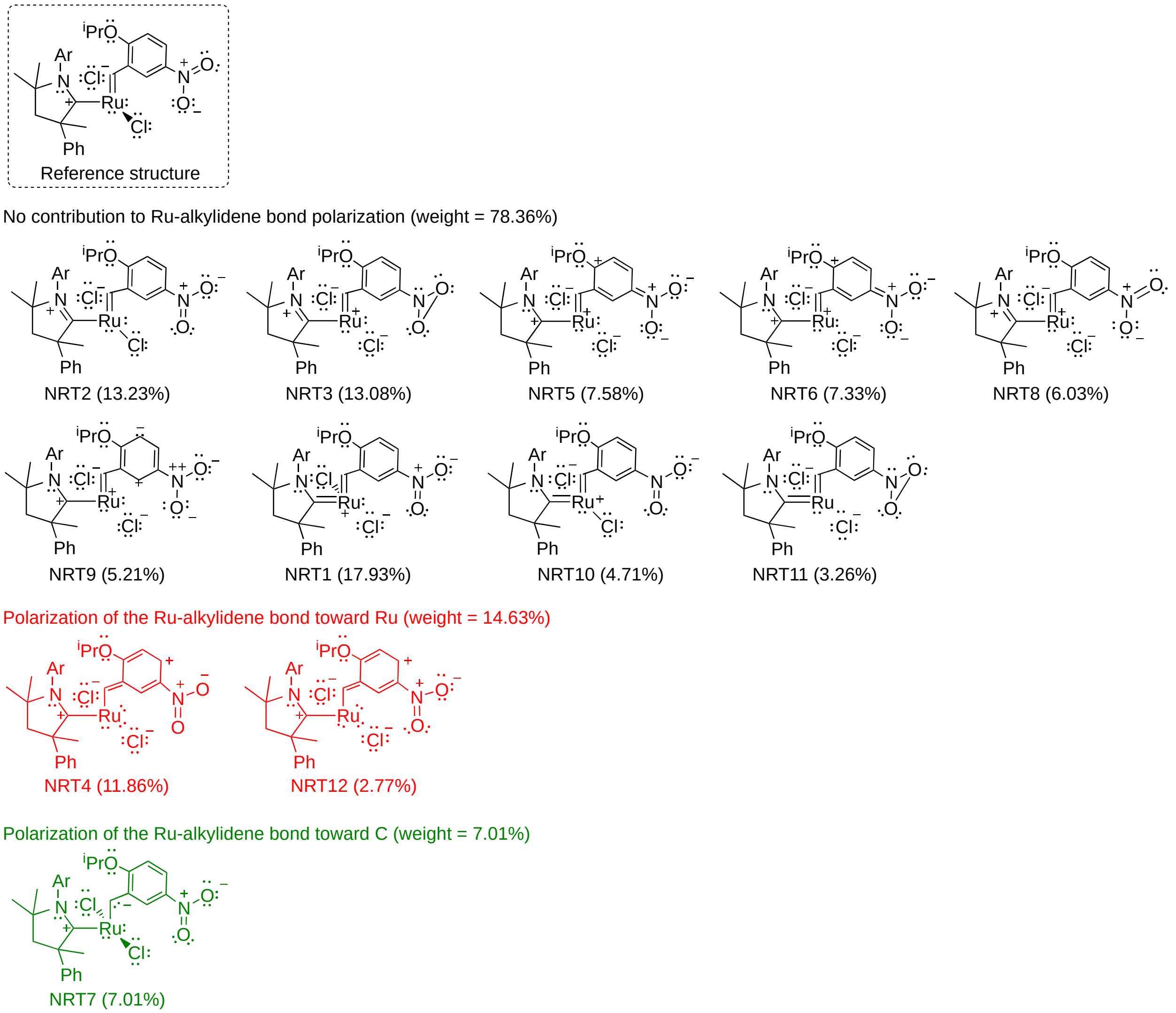

Figure S12. Weights and resonance structures from the single-reference NRT analysis of **2-nG-C1^{Ph}**, with the minimized NRT objective function (i.e., the root-mean-square deviation of the NRT one-electron reduced density matrix from the original DFT one-electron reduced density matrix, $\Delta(\{w\})$ = 0.04245298). Resonance forms of the substituents Ar (2,6-diethylphenyl), Ph (phenyl), and iPr (isopropyl) are omitted for clarity.

S2.2.2 Multi-Reference NRT analysis

To ensure a greater variety of resonance forms in the NRT analysis, we first generated resonance expansions using two references in each case: for **2-HC1^{Ph}**, those of Figure S10a and NRT8 (Figure S11); for **2-HC1^{Ph}**, those of Figures S10b and S10c. Structures NRT8 of **HC1^{Ph}** and that of Figure S10b of **2-nG-C1^{Ph}** both feature covalently bonded Cl atoms and Ru–alkylidene single bonds, and thus contribute to overlapping resonance-structure manifolds. Next, from these two-reference expansions, all resonance forms with a weight > 1% were specified as reference structures in the subsequent multi-reference NRT analyses. This strategy resulted 11 reference structures for **2-HC1^{Ph}** and 22 for **2-nG-C1^{Ph}**.

The corresponding multi-reference NRT analyses produced 15 resonance structures for **2-HC1^{Ph}** and 37 for **2-nG-C1^{Ph}**. In both cases, the minimized NRT objective function (the root-mean-square deviation of the NRT one-electron reduced density matrix from the original DFT one-electron reduced density matrix) $\Delta(\{w\})$ decreased by more than 0.002 compared to those obtained for the single-reference NRT analyses shown in Figures S11 and S12 above.

To facilitate analysis, selected structures are collected and simplified in Figure S13. Moreover, the complex picture emerging from a total of 52 resonance structures and their weights is most efficiently communicated by the corresponding natural bond orders (Table S2).

All 15 resonance structures of **2-HC1^{Ph}** are shown in Figure S14, and all those of **2-nG-C1^{Ph}** are shown in Figures S15 (structures with Ru–alkylidene double bond and one covalent Ru–Cl bond) and S16 (the remaining structures).

Table S2. Interatomic Distances of Selected Bonds and the Corresponding Natural Bond Orders of Single- (SR) and Multi-Reference (MR) NRT Analyses.[a]

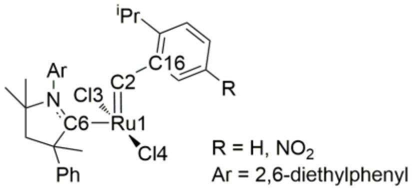

entry	bond	**2-HC1^{Ph}** NBO, SR NRT	**2-nG-C1^{Ph}** NBO, SR NRT	**2-HC1^{Ph}** NBO, MR NRT	**2-nG-C1^{Ph}** NBO, MR NRT	**2-HC1^{Ph}** (distance, Å)	**2-nG-C1^{Ph}** (distance, Å)
1	Ru1–C2	t =1.6640	t =1.7836	t =1.4872	t =1.5128	1.851	1.846
		c = 1.3158	c = 1.4534	c = 1.1786	c = 1.2086		
		i = 0.3482	i = 0.3302	i = 0.3086	i = 0.3041		
2	Ru1–Cl3	t = 0.3411	t =0.2494	t = 0.5566	t =0.6242	2.341	2.331
		c = 0.1621	c = 0.1277	c = 0.2978	c = 0.3443		
		i = 0.1790	i = 0.1218	i = 0.2587	i = 0.2798		
3	Ru1–Cl4	t =0.3489	t =0.2496	t =0.5619	t =0.6260	2.330	2.324
		c = 0.1659	c = 0.1280	c = 0.3177	c = 0.3431		
		i = 0.1830	i = 0.1216	i = 0.2442	i = 0.2829		
4	Ru1–C6	t =1.3062	t =1.2590	t =1.2380	t =1.1930	1.897	1.899
		c = 1.0829	c = 1.0573	c = 0.9926	c = 0.9585		
		i = 0.2232	i = 0.2018	i = 0.2454	i = 0.2345		
5	C2–C16	t =1.2055	t =1.1463	t =1.2940	t =1.1097	1.456	1.460
		c = 1.0607	c = 1.0039	c = 1.1274	c = 0.9851		
		i = 0.1447	i = 0.1424	i = 0.1666	i = 0.1246		

[a] Covalent natural bond order: "c". Ionic natural bond order: "i". Total natural bond order: "t".

The long-range polarizing effect of the nitro group manifests itself in modulated bond orders within bonds near the ruthenium center. Whereas some bond orders of **2-nG-C1^{Ph}** are lower than those of **HC1^{Ph}** (C2–C16 (by 0.18) and Ru1–C6 (by 0.05)), others are higher (Ru1–Cl3 (by 0.07), Ru1–Cl4 (by 0.06), and Ru1–C2 (by 0.02)).

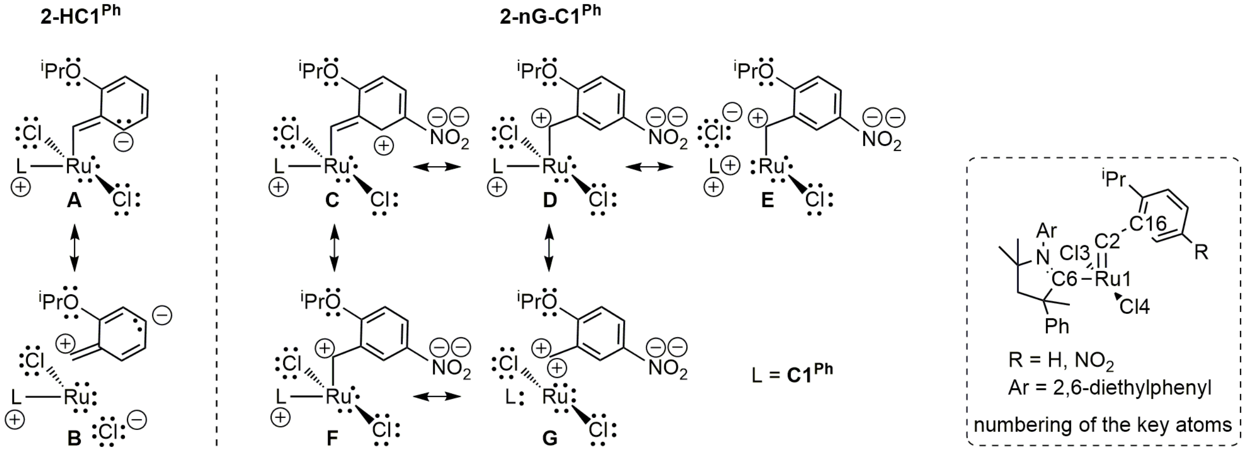

Figure S13. Selected, simplified resonance structures of the multi-reference NRT analyses of **2-HC1^{Ph}** and **2-nG-C1^{Ph}**, showing the long-range polarizating effects of the nitro group.

To illustrate how the differences in bond orders for **2-HC1^{Ph}** and **2-nG-C1^{Ph}** (Table S2) originate in mesomeric effects resulting from the nitro group, we now turn to two of the most important resonance forms of **2-HC1^{Ph}**, **A** and **B** (Figure S13). Resonance form **A** (weight = 18.4%) has a double bond between the alkylidene carbon atom and the aryl substituent (C2–C16): this resonance structure contributes most to the elevated C2–C16 bond order (1.29) in **2-HC1^{Ph}**. The corresponding resonance form in **2-nG-C1^{Ph}** is **C**, but the nitro group reduces its weight (10.4%) instead favouring **D** (9.9%) and **E** (13.1%), both of which have only C2–C16 single bonds. This shift toward resonance forms with a singly-bonded aryl group for **2-nG-C1^{Ph}** explains the reduced C2–C16 bond order (1.11) compared to that of **2-HC1^{Ph}** (1.29). Resonance form **E** also contributes to the lower Ru–**C1^{Ph}** bond order in **2-nG-C1^{Ph}** (1.19) vs **2-HC1^{Ph}** (1.24), by involving complete transfer of the carbene lone pair to ruthenium and thus not having a covalent Ru–**C1^{Ph}** bond.

Like **A**, resonance form **B** (4.3 %) has a C2–C16 double bond, contributes to the elevated bond order in **2-HC1^{Ph}**. This form also has an anionic chloride ligand and no alkylidene bond, and thus contributes to the decrease in the corresponding Ru–Cl and the Ru–alkylidene bond orders in **2-HC1^{Ph}**. Due to the nitro group, **2-nG-C1^{Ph}** has **F** (9.9 %) and **G** (5.8%) instead of **B**, and these forms feature C2–C16 single bonds and two covalent Ru–Cl bonds. Thus, **F** and **G** contribute to a decreased C2–C16 bond order and increased Ru–Cl bond orders in **2-nG-C1^{Ph}** vs **2-HC1^{Ph}**. Furthermore, whereas **B** has no alkylidene bond, **F** has a Ru1–C2 single bond, and thus contributes to a higher Ru–alkylidene bond order in **2-nG-C1^{Ph}** (1.51) vs **2-HC1^{Ph}** (1.49).

Like **E**, form **G** has no covalent Ru–**C1^{Ph}** bond. The combined weight of **E** and **G** is 21.0%, whereas the overall weight of the resonance forms of **HC1^{Ph}** without Ru–**C1^{Ph}** bonds is lower (16.7%). This difference (4.7 percentage points), which is caused by the long-range polarization effects of the nitro group, explains the lower Ru–**C1^{Ph}** bond order in **2-nG-C1^{Ph}** (1.19) vs **2-HC1^{Ph}** (1.24).

In summary, the above resonance forms and weights illustrate how the nitro group reduces the Ru–**C1^{Ph}** bond order and increases the Ru–Cl and Ru–alkylidene bond orders, as seen in Table S2.

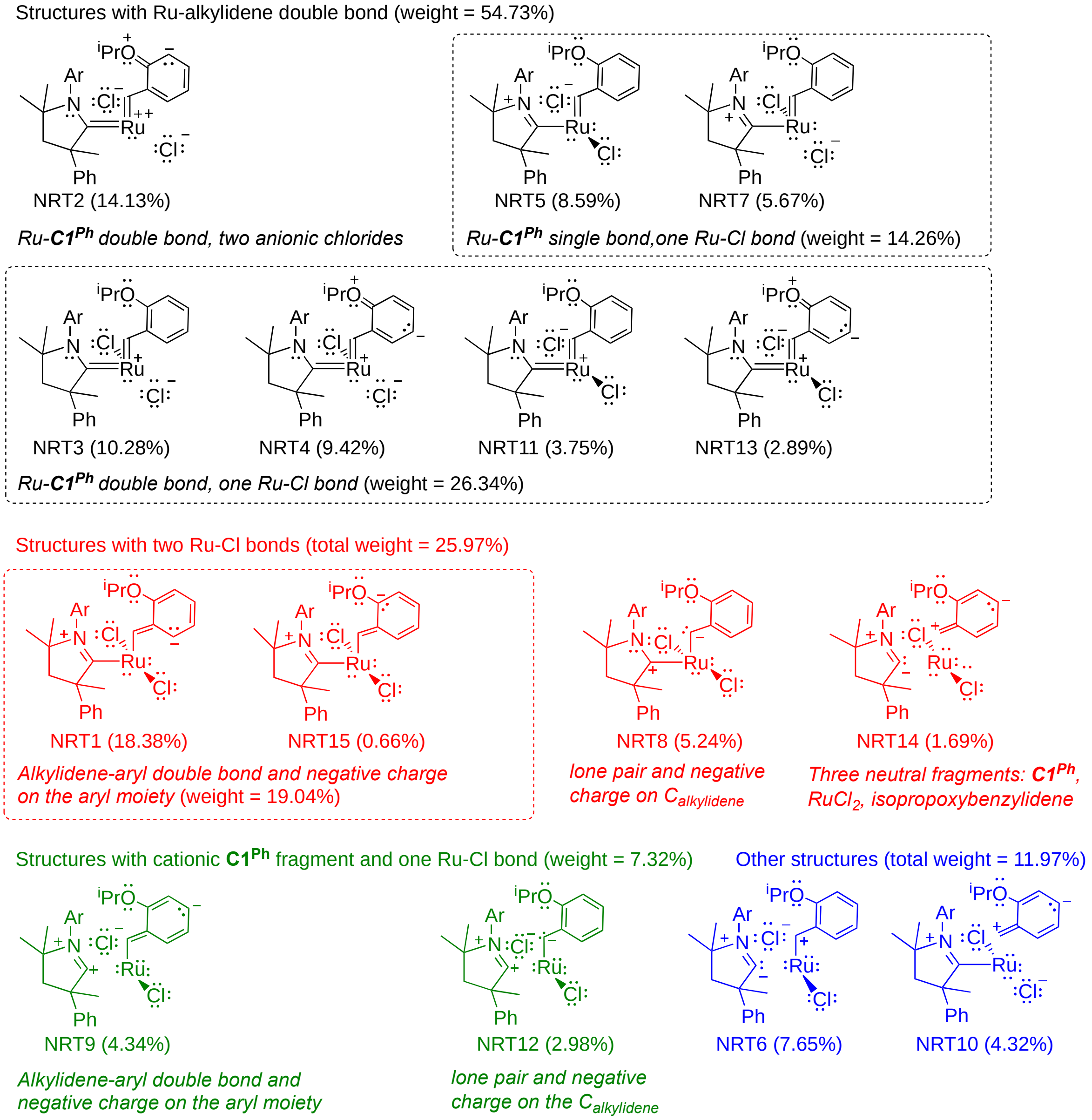

Figure S14. Resonance structures and weights of the multi-reference NRT analysis of **2-HC1^{Ph}**, with the minimized NRT objective function (i.e., the root-mean-square deviation of the NRT one-electron reduced density matrix from the original DFT one-electron reduced density matrix, $\Delta(\{w\})$ = 0.03918091. Resonance forms of the substituents Ar (2,6-diethylphenyl), Ph (phenyl), and iPr (isopropyl) are omitted for clarity.

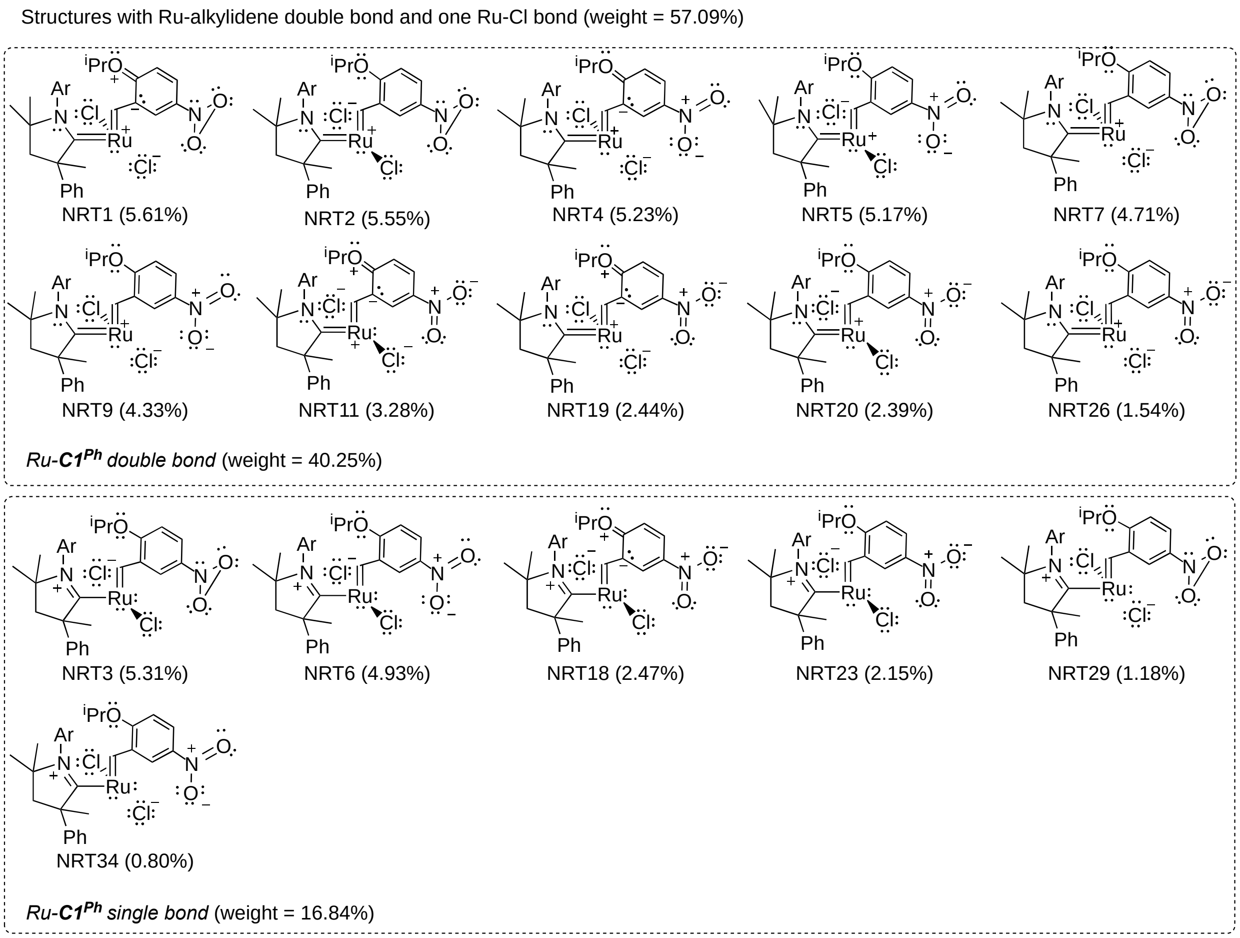

Figure S15. Multi-reference NRT analysis of **2-nG-C1^{Ph}**: resonance structures (and weights) with Ru–alkylidene double bond and one covalent Ru–Cl bond, with the minimized NRT objective function (i.e., the root-mean-square deviation of the NRT one-electron reduced density matrix from the original DFT one-electron reduced density matrix, $\Delta(\{w\})$ = 0.04022816). Resonance forms of the substituents Ar (2,6-diethylphenyl), Ph (phenyl), and iPr (isopropyl) are omitted for clarity.

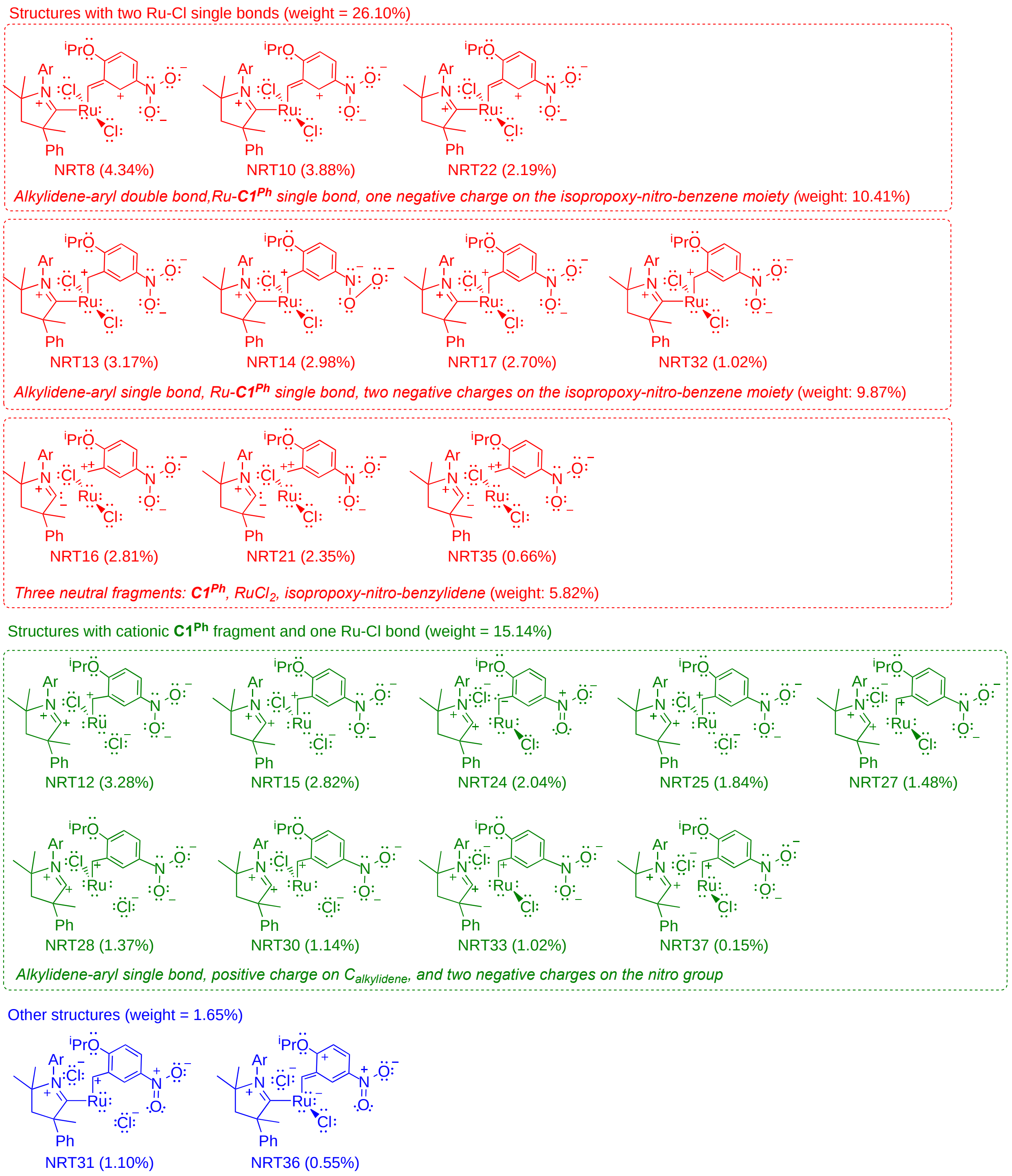

Figure S16. Multi-reference NRT analysis of **2-nGC1^{Ph}**: resonance structures (and weights) with two covalent Ru–Cl bonds, one covalent Ru–Cl bond and a cationic **C1^{Ph}** fragment, and other structures, respectively. See Figure S15 for structures with Ru–alkylidene double bond and one covalent Ru–Cl bond, with the minimized NRT objective function (i.e., the root-mean-square deviation of the NRT one-electron reduced density matrix from the original DFT one-electron reduced density matrix, $\Delta(\{w\})$ = 0.04022816). Resonance forms of the substituents Ar (2,6-diethylphenyl), Ph (phenyl), and iPr (isopropyl) are omitted for clarity.

S2.2.3 Input File for NRT Analysis of 2-HC1^{Ph}

The first part of the GenNBO input file, including program options and the definitions of the 11 reference structures, used for the multi-reference NRT analysis of **2-HC1^{Ph}** is given below, with the corresponding atom enumeration in Figure S17.

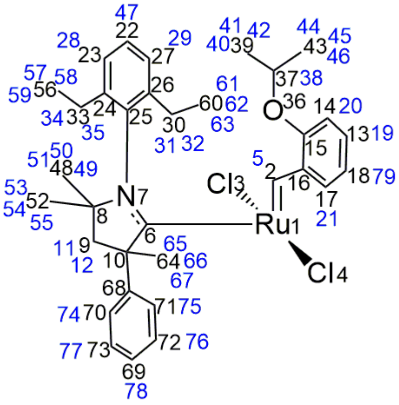

Figure S17. Atom enumeration for **2-HC1^{Ph}**. Hydrogen atoms are omitted for clarity: their numbers (in blue) are indicated next to the carbon atoms to which they are bonded.

```
$GENNBO  NATOMS=79  NBAS=3472  UPPER  BODM  FORMAT=PRECISE  $END
 $NBO MEMORY=2048mb NRTMEM=5 NRTTHR=20 NRTLST=1 FILE=2-HC1Ph_mr $END
 $NRTSTR
   STR1
     LONE 1 2 3 3 4 3 7 1 13 1 36 2 END
     BOND S 1 2 S 1 3 S 1 4 S 1 6 S 2 5 D 2 16 S 6 7 S 6 10 S 7 8 S 7 25
          S 8 9 S 8 48 S 8 52 S 9 10 S 9 11 S 9 12 S 10 64 S 10 68 S 13 14
          S 13 18 S 13 19 D 14 15 S 14 20 S 15 16 S 15 36 S 16 17 D 17 18
          S 17 21 S 18 79 S 22 23 D 22 27 S 22 47 D 23 24 S 23 28 S 24 25
          S 24 33 D 25 26 S 26 27 S 26 30 S 27 29 S 30 31 S 30 32 S 30 60
          S 33 34 S 33 35 S 33 56 S 36 37 S 37 38 S 37 39 S 37 43 S 39 40
          S 39 41 S 39 42 S 43 44 S 43 45 S 43 46 S 48 49 S 48 50 S 48 51
          S 52 53 S 52 54 S 52 55 S 56 57 S 56 58 S 56 59 S 60 61 S 60 62
          S 60 63 S 64 65 S 64 66 S 64 67 S 68 70 D 68 71 D 69 72 S 69 73
          S 69 78 D 70 73 S 70 74 S 71 72 S 71 75 S 72 76 S 73 77 END
   END
   STR2
     LONE 1 1 3 3 4 4 7 1 36 2 END
     BOND D 1 2 S 1 3 D 1 6 S 2 5 S 2 16 S 6 7 S 6 10 S 7 8 S 7 25 S 8 9
          S 8 48 S 8 52 S 9 10 S 9 11 S 9 12 S 10 64 S 10 68 D 13 14 S 13 18
          S 13 19 S 14 15 S 14 20 D 15 16 S 15 36 S 16 17 D 17 18 S 17 21
          S 18 79 S 22 23 D 22 27 S 22 47 D 23 24 S 23 28 S 24 25 S 24 33
          D 25 26 S 26 27 S 26 30 S 27 29 S 30 31 S 30 32 S 30 60 S 33 34
          S 33 35 S 33 56 S 36 37 S 37 38 S 37 39 S 37 43 S 39 40 S 39 41
          S 39 42 S 43 44 S 43 45 S 43 46 S 48 49 S 48 50 S 48 51 S 52 53
          S 52 54 S 52 55 S 56 57 S 56 58 S 56 59 S 60 61 S 60 62 S 60 63
          S 64 65 S 64 66 S 64 67 S 68 70 D 68 71 D 69 72 S 69 73 S 69 78
          D 70 73 S 70 74 S 71 72 S 71 75 S 72 76 S 73 77 END
   END
   STR3
     LONE 1 1 3 4 4 4 7 1 16 1 36 1 END
     BOND D 1 2 D 1 6 S 2 5 S 2 16 S 6 7 S 6 10 S 7 8 S 7 25 S 8 9 S 8 48
          S 8 52 S 9 10 S 9 11 S 9 12 S 10 64 S 10 68 D 13 14 S 13 18 S 13 19
          S 14 15 S 14 20 S 15 16 D 15 36 S 16 17 D 17 18 S 17 21 S 18 79
          S 22 23 D 22 27 S 22 47 D 23 24 S 23 28 S 24 25 S 24 33 D 25 26
          S 26 27 S 26 30 S 27 29 S 30 31 S 30 32 S 30 60 S 33 34 S 33 35
          S 33 56 S 36 37 S 37 38 S 37 39 S 37 43 S 39 40 S 39 41 S 39 42
          S 43 44 S 43 45 S 43 46 S 48 49 S 48 50 S 48 51 S 52 53 S 52 54
          S 52 55 S 56 57 S 56 58 S 56 59 S 60 61 S 60 62 S 60 63 S 64 65
          S 64 66 S 64 67 S 68 70 D 68 71 D 69 72 S 69 73 S 69 78 D 70 73
          S 70 74 S 71 72 S 71 75 S 72 76 S 73 77 END
   END
```

```
STR4
  LONE 1 2 3 4 4 3 36 2 END
  BOND D 1 2 S 1 4 S 1 6 S 2 5 S 2 16 D 6 7 S 6 10 S 7 8 S 7 25 S 8 9
       S 8 48 S 8 52 S 9 10 S 9 11 S 9 12 S 10 64 S 10 68 D 13 14 S 13 18
       S 13 19 S 14 15 S 14 20 D 15 16 S 15 36 S 16 17 D 17 18 S 17 21
       S 18 79 S 22 23 D 22 27 S 22 47 D 23 24 S 23 28 S 24 25 S 24 33
       D 25 26 S 26 27 S 26 30 S 27 29 S 30 31 S 30 32 S 30 60 S 33 34
       S 33 35 S 33 56 S 36 37 S 37 38 S 37 39 S 37 43 S 39 40 S 39 41
       S 39 42 S 43 44 S 43 45 S 43 46 S 48 49 S 48 50 S 48 51 S 52 53
       S 52 54 S 52 55 S 56 57 S 56 58 S 56 59 S 60 61 S 60 62 S 60 63
       S 64 65 S 64 66 S 64 67 S 68 70 D 68 71 D 69 72 S 69 73 S 69 78
       D 70 73 S 70 74 S 71 72 S 71 75 S 72 76 S 73 77 END
END
STR5
  LONE 1 2 3 3 4 3 7 1 15 1 18 1 36 2 END
  BOND S 1 2 S 1 3 S 1 4 S 1 6 S 2 5 S 2 16 S 6 7 S 6 10 S 7 8 S 7 25
       S 8 9 S 8 48 S 8 52 S 9 10 S 9 11 S 9 12 S 10 64 S 10 68 D 13 14
       S 13 18 S 13 19 S 14 15 S 14 20 S 15 16 S 15 36 D 16 17 S 17 18
       S 17 21 S 18 79 S 22 23 D 22 27 S 22 47 D 23 24 S 23 28 S 24 25
       S 24 33 D 25 26 S 26 27 S 26 30 S 27 29 S 30 31 S 30 32 S 30 60
       S 33 34 S 33 35 S 33 56 S 36 37 S 37 38 S 37 39 S 37 43 S 39 40
       S 39 41 S 39 42 S 43 44 S 43 45 S 43 46 S 48 49 S 48 50 S 48 51
       S 52 53 S 52 54 S 52 55 S 56 57 S 56 58 S 56 59 S 60 61 S 60 62
       S 60 63 S 64 65 S 64 66 S 64 67 S 68 70 D 68 71 D 69 72 S 69 73
       S 69 78 D 70 73 S 70 74 S 71 72 S 71 75 S 72 76 S 73 77 END
END
STR6
  LONE 1 2 3 3 4 4 36 2 END
  BOND D 1 2 S 1 3 S 1 6 S 2 5 S 2 16 D 6 7 S 6 10 S 7 8 S 7 25 S 8 9
       S 8 48 S 8 52 S 9 10 S 9 11 S 9 12 S 10 64 S 10 68 D 13 14 S 13 18
       S 13 19 S 14 15 S 14 20 D 15 16 S 15 36 S 16 17 D 17 18 S 17 21
       S 18 79 S 22 23 D 22 27 S 22 47 D 23 24 S 23 28 S 24 25 S 24 33
       D 25 26 S 26 27 S 26 30 S 27 29 S 30 31 S 30 32 S 30 60 S 33 34
       S 33 35 S 33 56 S 36 37 S 37 38 S 37 39 S 37 43 S 39 40 S 39 41
       S 39 42 S 43 44 S 43 45 S 43 46 S 48 49 S 48 50 S 48 51 S 52 53
       S 52 54 S 52 55 S 56 57 S 56 58 S 56 59 S 60 61 S 60 62 S 60 63
       S 64 65 S 64 66 S 64 67 S 68 70 D 68 71 D 69 72 S 69 73 S 69 78
       D 70 73 S 70 74 S 71 72 S 71 75 S 72 76 S 73 77 END
END
STR7
  LONE 1 3 2 1 3 4 4 3 36 2 END
  BOND S 1 2 S 1 4 S 2 5 S 2 16 D 6 7 S 6 10 S 7 8 S 7 25 S 8 9 S 8 48
       S 8 52 S 9 10 S 9 11 S 9 12 S 10 64 S 10 68 D 13 14 S 13 18 S 13 19
       S 14 15 S 14 20 D 15 16 S 15 36 S 16 17 D 17 18 S 17 21 S 18 79
       S 22 23 D 22 27 S 22 47 D 23 24 S 23 28 S 24 25 S 24 33 D 25 26
       S 26 27 S 26 30 S 27 29 S 30 31 S 30 32 S 30 60 S 33 34 S 33 35
       S 33 56 S 36 37 S 37 38 S 37 39 S 37 43 S 39 40 S 39 41 S 39 42
       S 43 44 S 43 45 S 43 46 S 48 49 S 48 50 S 48 51 S 52 53 S 52 54
       S 52 55 S 56 57 S 56 58 S 56 59 S 60 61 S 60 62 S 60 63 S 64 65
       S 64 66 S 64 67 S 68 70 D 68 71 D 69 72 S 69 73 S 69 78 D 70 73
       S 70 74 S 71 72 S 71 75 S 72 76 S 73 77 END
END
STR8
  LONE 1 3 3 4 4 4 36 2 END
  BOND S 1 2 S 1 6 S 2 5 D 2 16 D 6 7 S 6 10 S 7 8 S 7 25 S 8 9 S 8 48
       S 8 52 S 9 10 S 9 11 S 9 12 S 10 64 S 10 68 D 13 14 S 13 18 S 13 19
       S 14 15 S 14 20 S 15 16 S 15 36 S 16 17 D 17 18 S 17 21 S 18 79
       S 22 23 D 22 27 S 22 47 D 23 24 S 23 28 S 24 25 S 24 33 D 25 26
       S 26 27 S 26 30 S 27 29 S 30 31 S 30 32 S 30 60 S 33 34 S 33 35
       S 33 56 S 36 37 S 37 38 S 37 39 S 37 43 S 39 40 S 39 41 S 39 42
       S 43 44 S 43 45 S 43 46 S 48 49 S 48 50 S 48 51 S 52 53 S 52 54
       S 52 55 S 56 57 S 56 58 S 56 59 S 60 61 S 60 62 S 60 63 S 64 65
       S 64 66 S 64 67 S 68 70 D 68 71 D 69 72 S 69 73 S 69 78 D 70 73
       S 70 74 S 71 72 S 71 75 S 72 76 S 73 77 END
END
STR9
  LONE 1 3 2 1 3 4 4 3 36 2 END
  BOND S 1 2 S 1 4 S 2 5 S 2 16 D 6 7 S 6 10 S 7 8 S 7 25 S 8 9 S 8 48
       S 8 52 S 9 10 S 9 11 S 9 12 S 10 64 S 10 68 D 13 14 S 13 18 S 13 19
       S 14 15 S 14 20 D 15 16 S 15 36 S 16 17 D 17 18 S 17 21 S 18 79
       S 22 23 D 22 27 S 22 47 D 23 24 S 23 28 S 24 25 S 24 33 D 25 26
       S 26 27 S 26 30 S 27 29 S 30 31 S 30 32 S 30 60 S 33 34 S 33 35
       S 33 56 S 36 37 S 37 38 S 37 39 S 37 43 S 39 40 S 39 41 S 39 42
```

```
          S 43 44 S 43 45 S 43 46 S 48 49 S 48 50 S 48 51 S 52 53 S 52 54
          S 52 55 S 56 57 S 56 58 S 56 59 S 60 61 S 60 62 S 60 63 S 64 65
          S 64 66 S 64 67 S 68 70 D 68 71 D 69 72 S 69 73 S 69 78 D 70 73
          S 70 74 S 71 72 S 71 75 S 72 76 S 73 77 END
   END
   STR10
     LONE 1 2 3 3 4 3 15 1 36 2 END
     BOND S 1 2 S 1 3 S 1 4 S 1 6 S 2 5 D 2 16 D 6 7 S 6 10 S 7 8 S 7 25
          S 8 9 S 8 48 S 8 52 S 9 10 S 9 11 S 9 12 S 10 64 S 10 68 D 13 14
          S 13 18 S 13 19 S 14 15 S 14 20 S 15 16 S 15 36 S 16 17 D 17 18
          S 17 21 S 18 79 S 22 23 D 22 27 S 22 47 D 23 24 S 23 28 S 24 25
          S 24 33 D 25 26 S 26 27 S 26 30 S 27 29 S 30 31 S 30 32 S 30 60
          S 33 34 S 33 35 S 33 56 S 36 37 S 37 38 S 37 39 S 37 43 S 39 40
          S 39 41 S 39 42 S 43 44 S 43 45 S 43 46 S 48 49 S 48 50 S 48 51
          S 52 53 S 52 54 S 52 55 S 56 57 S 56 58 S 56 59 S 60 61 S 60 62
          S 60 63 S 64 65 S 64 66 S 64 67 S 68 70 D 68 71 D 69 72 S 69 73
          S 69 78 D 70 73 S 70 74 S 71 72 S 71 75 S 72 76 S 73 77 END
   END
   STR11
     LONE 1 1 3 4 4 3 7 1 36 2 END
     BOND D 1 2 S 1 4 D 1 6 S 2 5 S 2 16 S 6 7 S 6 10 S 7 8 S 7 25 S 8 9
          S 8 48 S 8 52 S 9 10 S 9 11 S 9 12 S 10 64 S 10 68 D 13 14 S 13 18
          S 13 19 S 14 15 S 14 20 D 15 16 S 15 36 S 16 17 D 17 18 S 17 21
          S 18 79 S 22 23 D 22 27 S 22 47 D 23 24 S 23 28 S 24 25 S 24 33
          D 25 26 S 26 27 S 26 30 S 27 29 S 30 31 S 30 32 S 30 60 S 33 34
          S 33 35 S 33 56 S 36 37 S 37 38 S 37 39 S 37 43 S 39 40 S 39 41
          S 39 42 S 43 44 S 43 45 S 43 46 S 48 49 S 48 50 S 48 51 S 52 53
          S 52 54 S 52 55 S 56 57 S 56 58 S 56 59 S 60 61 S 60 62 S 60 63
          S 64 65 S 64 66 S 64 67 S 68 70 D 68 71 D 69 72 S 69 73 S 69 78
          D 70 73 S 70 74 S 71 72 S 71 75 S 72 76 S 73 77 END
   END
 $END
```

S2.2.4 Input File for NRT Analysis of 2-nG-C1^{Ph}

The first part of the GenNBO input file, including program options and the definitions of the 22 reference structures, used for the multi-reference NRT analysis of **2-HC1^{Ph}** is given below, with the corresponding atom enumeration in Figure S18.

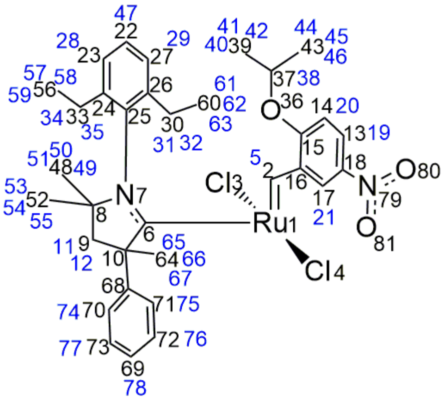

Figure S18. Atom enumeration of **2-nG-C1^{Ph}**. Hydrogen atoms are omitted for clarity, but their numbers (in blue) are indicated next to the carbon atoms to which they are bonded.

```
$GENNBO  NATOMS=81  NBAS=3682  UPPER  BODM  FORMAT=PRECISE  $END
 $NBO MEMORY=2048mb NRTMEM=5 NRTTHR=20 NRTLST=1 FILE=2-nG-C1Ph_mr $END
 $NRTSTR
   STR1
     LONE 1 1 3 4 4 4 7 1 36 2 79 1 80 2 81 2 END
     BOND D 1 2 D 1 6 S 2 5 S 2 16 S 6 7 S 6 10 S 7 8 S 7 25 S 8 9 S 8 48
```

```
       S 8 52 S 9 10 S 9 11 S 9 12 S 10 64 S 10 68 D 13 14 S 13 18 S 13 19
       S 14 15 S 14 20 D 15 16 S 15 36 S 16 17 D 17 18 S 17 21 S 18 79
       S 22 23 D 22 27 S 22 47 D 23 24 S 23 28 S 24 25 S 24 33 D 25 26
       S 26 27 S 26 30 S 27 29 S 30 31 S 30 32 S 30 60 S 33 34 S 33 35
       S 33 56 S 36 37 S 37 38 S 37 39 S 37 43 S 39 40 S 39 41 S 39 42
       S 43 44 S 43 45 S 43 46 S 48 49 S 48 50 S 48 51 S 52 53 S 52 54
       S 52 55 S 56 57 S 56 58 S 56 59 S 60 61 S 60 62 S 60 63 S 64 65
       S 64 66 S 64 67 S 68 70 D 68 71 D 69 72 S 69 73 S 69 78 D 70 73
       S 70 74 S 71 72 S 71 75 S 72 76 S 73 77 S 79 80 S 79 81 S 80 81 END
END
STR2
  LONE 1 1 3 3 4 4 7 1 36 2 80 3 81 2 END
  BOND D 1 2 S 1 3 D 1 6 S 2 5 S 2 16 S 6 7 S 6 10 S 7 8 S 7 25 S 8 9
       S 8 48 S 8 52 S 9 10 S 9 11 S 9 12 S 10 64 S 10 68 D 13 14 S 13 18
       S 13 19 S 14 15 S 14 20 D 15 16 S 15 36 S 16 17 D 17 18 S 17 21
       S 18 79 S 22 23 D 22 27 S 22 47 D 23 24 S 23 28 S 24 25 S 24 33
       D 25 26 S 26 27 S 26 30 S 27 29 S 30 31 S 30 32 S 30 60 S 33 34
       S 33 35 S 33 56 S 36 37 S 37 38 S 37 39 S 37 43 S 39 40 S 39 41
       S 39 42 S 43 44 S 43 45 S 43 46 S 48 49 S 48 50 S 48 51 S 52 53
       S 52 54 S 52 55 S 56 57 S 56 58 S 56 59 S 60 61 S 60 62 S 60 63
       S 64 65 S 64 66 S 64 67 S 68 70 D 68 71 D 69 72 S 69 73 S 69 78
       D 70 73 S 70 74 S 71 72 S 71 75 S 72 76 S 73 77 S 79 80 D 79 81 END
END
STR3
  LONE 1 1 3 4 4 3 7 1 36 2 80 3 81 2 END
  BOND D 1 2 S 1 4 D 1 6 S 2 5 S 2 16 S 6 7 S 6 10 S 7 8 S 7 25 S 8 9
       S 8 48 S 8 52 S 9 10 S 9 11 S 9 12 S 10 64 S 10 68 D 13 14 S 13 18
       S 13 19 S 14 15 S 14 20 D 15 16 S 15 36 S 16 17 D 17 18 S 17 21
       S 18 79 S 22 23 D 22 27 S 22 47 D 23 24 S 23 28 S 24 25 S 24 33
       D 25 26 S 26 27 S 26 30 S 27 29 S 30 31 S 30 32 S 30 60 S 33 34
       S 33 35 S 33 56 S 36 37 S 37 38 S 37 39 S 37 43 S 39 40 S 39 41
       S 39 42 S 43 44 S 43 45 S 43 46 S 48 49 S 48 50 S 48 51 S 52 53
       S 52 54 S 52 55 S 56 57 S 56 58 S 56 59 S 60 61 S 60 62 S 60 63
       S 64 65 S 64 66 S 64 67 S 68 70 D 68 71 D 69 72 S 69 73 S 69 78
       D 70 73 S 70 74 S 71 72 S 71 75 S 72 76 S 73 77 S 79 80 D 79 81 END
END
STR4
  LONE 1 1 3 4 4 4 7 1 36 2 80 2 81 3 END
  BOND D 1 2 D 1 6 S 2 5 S 2 16 S 6 7 S 6 10 S 7 8 S 7 25 S 8 9 S 8 48
       S 8 52 S 9 10 S 9 11 S 9 12 S 10 64 S 10 68 D 13 14 S 13 18 S 13 19
       S 14 15 S 14 20 D 15 16 S 15 36 S 16 17 D 17 18 S 17 21 S 18 79
       S 22 23 D 22 27 S 22 47 D 23 24 S 23 28 S 24 25 S 24 33 D 25 26
       S 26 27 S 26 30 S 27 29 S 30 31 S 30 32 S 30 60 S 33 34 S 33 35
       S 33 56 S 36 37 S 37 38 S 37 39 S 37 43 S 39 40 S 39 41 S 39 42
       S 43 44 S 43 45 S 43 46 S 48 49 S 48 50 S 48 51 S 52 53 S 52 54
       S 52 55 S 56 57 S 56 58 S 56 59 S 60 61 S 60 62 S 60 63 S 64 65
       S 64 66 S 64 67 S 68 70 D 68 71 D 69 72 S 69 73 S 69 78 D 70 73
       S 70 74 S 71 72 S 71 75 S 72 76 S 73 77 D 79 80 S 79 81 END
END
STR5
  LONE 1 2 3 3 4 3 36 2 79 1 80 3 81 3 END
  BOND S 1 2 S 1 3 S 1 4 S 1 6 S 2 5 D 2 16 D 6 7 S 6 10 S 7 8 S 7 25
       S 8 9 S 8 48 S 8 52 S 9 10 S 9 11 S 9 12 S 10 64 S 10 68 S 13 14
       D 13 18 S 13 19 D 14 15 S 14 20 S 15 16 S 15 36 S 16 17 S 17 18
       S 17 21 S 18 79 S 22 23 D 22 27 S 22 47 D 23 24 S 23 28 S 24 25
       S 24 33 D 25 26 S 26 27 S 26 30 S 27 29 S 30 31 S 30 32 S 30 60
       S 33 34 S 33 35 S 33 56 S 36 37 S 37 38 S 37 39 S 37 43 S 39 40
       S 39 41 S 39 42 S 43 44 S 43 45 S 43 46 S 48 49 S 48 50 S 48 51
       S 52 53 S 52 54 S 52 55 S 56 57 S 56 58 S 56 59 S 60 61 S 60 62
       S 60 63 S 64 65 S 64 66 S 64 67 S 68 70 D 68 71 D 69 72 S 69 73
       S 69 78 D 70 73 S 70 74 S 71 72 S 71 75 S 72 76 S 73 77 S 79 80
       S 79 81 END
END
STR6
  LONE 1 2 3 3 4 3 36 2 79 2 80 3 81 2 END
  BOND S 1 2 S 1 3 S 1 4 S 1 6 S 2 5 S 2 16 D 6 7 S 6 10 S 7 8 S 7 25
       S 8 9 S 8 48 S 8 52 S 9 10 S 9 11 S 9 12 S 10 64 S 10 68 S 13 14
       D 13 18 S 13 19 D 14 15 S 14 20 S 15 16 S 15 36 D 16 17 S 17 18
       S 17 21 S 18 79 S 22 23 D 22 27 S 22 47 D 23 24 S 23 28 S 24 25
       S 24 33 D 25 26 S 26 27 S 26 30 S 27 29 S 30 31 S 30 32 S 30 60
       S 33 34 S 33 35 S 33 56 S 36 37 S 37 38 S 37 39 S 37 43 S 39 40
       S 39 41 S 39 42 S 43 44 S 43 45 S 43 46 S 48 49 S 48 50 S 48 51
       S 52 53 S 52 54 S 52 55 S 56 57 S 56 58 S 56 59 S 60 61 S 60 62
```

```
       S 60 63 S 64 65 S 64 66 S 64 67 S 68 70 D 68 71 D 69 72 S 69 73
       S 69 78 D 70 73 S 70 74 S 71 72 S 71 75 S 72 76 S 73 77 S 79 81
       S 80 81 END
END
STR7
  LONE 1 2 3 3 4 3 36 2 79 1 80 3 81 3 END
  BOND S 1 2 S 1 3 S 1 4 S 1 6 S 2 5 D 2 16 D 6 7 S 6 10 S 7 8 S 7 25
       S 8 9 S 8 48 S 8 52 S 9 10 S 9 11 S 9 12 S 10 64 S 10 68 S 13 14
       D 13 18 S 13 19 D 14 15 S 14 20 S 15 16 S 15 36 S 16 17 S 17 18
       S 17 21 S 18 79 S 22 23 D 22 27 S 22 47 D 23 24 S 23 28 S 24 25
       S 24 33 D 25 26 S 26 27 S 26 30 S 27 29 S 30 31 S 30 32 S 30 60
       S 33 34 S 33 35 S 33 56 S 36 37 S 37 38 S 37 39 S 37 43 S 39 40
       S 39 41 S 39 42 S 43 44 S 43 45 S 43 46 S 48 49 S 48 50 S 48 51
       S 52 53 S 52 54 S 52 55 S 56 57 S 56 58 S 56 59 S 60 61 S 60 62
       S 60 63 S 64 65 S 64 66 S 64 67 S 68 70 D 68 71 D 69 72 S 69 73
       S 69 78 D 70 73 S 70 74 S 71 72 S 71 75 S 72 76 S 73 77 S 79 80
       S 79 81 END
END
STR8
  LONE 1 2 3 3 4 3 36 2 79 1 80 3 81 3 END
  BOND S 1 2 S 1 3 S 1 4 S 1 6 S 2 5 D 2 16 D 6 7 S 6 10 S 7 8 S 7 25
       S 8 9 S 8 48 S 8 52 S 9 10 S 9 11 S 9 12 S 10 64 S 10 68 S 13 14
       D 13 18 S 13 19 D 14 15 S 14 20 S 15 16 S 15 36 S 16 17 S 17 18
       S 17 21 S 18 79 S 22 23 D 22 27 S 22 47 D 23 24 S 23 28 S 24 25
       S 24 33 D 25 26 S 26 27 S 26 30 S 27 29 S 30 31 S 30 32 S 30 60
       S 33 34 S 33 35 S 33 56 S 36 37 S 37 38 S 37 39 S 37 43 S 39 40
       S 39 41 S 39 42 S 43 44 S 43 45 S 43 46 S 48 49 S 48 50 S 48 51
       S 52 53 S 52 54 S 52 55 S 56 57 S 56 58 S 56 59 S 60 61 S 60 62
       S 60 63 S 64 65 S 64 66 S 64 67 S 68 70 D 68 71 D 69 72 S 69 73
       S 69 78 D 70 73 S 70 74 S 71 72 S 71 75 S 72 76 S 73 77 S 79 80
       S 79 81 END
END
STR9
  LONE 1 2 3 3 4 3 14 1 36 1 79 1 80 3 81 3 END
  BOND S 1 2 S 1 3 S 1 4 S 1 6 S 2 5 S 2 16 D 6 7 S 6 10 S 7 8 S 7 25
       S 8 9 S 8 48 S 8 52 S 9 10 S 9 11 S 9 12 S 10 64 S 10 68 S 13 14
       D 13 18 S 13 19 S 14 15 S 14 20 S 15 16 D 15 36 D 16 17 S 17 18
       S 17 21 S 18 79 S 22 23 D 22 27 S 22 47 D 23 24 S 23 28 S 24 25
       S 24 33 D 25 26 S 26 27 S 26 30 S 27 29 S 30 31 S 30 32 S 30 60
       S 33 34 S 33 35 S 33 56 S 36 37 S 37 38 S 37 39 S 37 43 S 39 40
       S 39 41 S 39 42 S 43 44 S 43 45 S 43 46 S 48 49 S 48 50 S 48 51
       S 52 53 S 52 54 S 52 55 S 56 57 S 56 58 S 56 59 S 60 61 S 60 62
       S 60 63 S 64 65 S 64 66 S 64 67 S 68 70 D 68 71 D 69 72 S 69 73
       S 69 78 D 70 73 S 70 74 S 71 72 S 71 75 S 72 76 S 73 77 S 79 80
       S 79 81 END
END
STR10
  LONE 1 2 3 3 4 3 14 1 36 1 79 1 80 3 81 3 END
  BOND S 1 2 S 1 3 S 1 4 S 1 6 S 2 5 S 2 16 D 6 7 S 6 10 S 7 8 S 7 25
       S 8 9 S 8 48 S 8 52 S 9 10 S 9 11 S 9 12 S 10 64 S 10 68 S 13 14
       D 13 18 S 13 19 S 14 15 S 14 20 S 15 16 D 15 36 D 16 17 S 17 18
       S 17 21 S 18 79 S 22 23 D 22 27 S 22 47 D 23 24 S 23 28 S 24 25
       S 24 33 D 25 26 S 26 27 S 26 30 S 27 29 S 30 31 S 30 32 S 30 60
       S 33 34 S 33 35 S 33 56 S 36 37 S 37 38 S 37 39 S 37 43 S 39 40
       S 39 41 S 39 42 S 43 44 S 43 45 S 43 46 S 48 49 S 48 50 S 48 51
       S 52 53 S 52 54 S 52 55 S 56 57 S 56 58 S 56 59 S 60 61 S 60 62
       S 60 63 S 64 65 S 64 66 S 64 67 S 68 70 D 68 71 D 69 72 S 69 73
       S 69 78 D 70 73 S 70 74 S 71 72 S 71 75 S 72 76 S 73 77 S 79 80
       S 79 81 END
END
STR11
  LONE 1 3 3 4 4 3 36 2 79 1 80 3 81 3 END
  BOND S 1 2 S 1 4 S 2 5 S 2 16 D 6 7 S 6 10 S 7 8 S 7 25 S 8 9 S 8 48
       S 8 52 S 9 10 S 9 11 S 9 12 S 10 64 S 10 68 S 13 14 D 13 18 S 13 19
       D 14 15 S 14 20 S 15 16 S 15 36 D 16 17 S 17 18 S 17 21 S 18 79
       S 22 23 D 22 27 S 22 47 D 23 24 S 23 28 S 24 25 S 24 33 D 25 26
       S 26 27 S 26 30 S 27 29 S 30 31 S 30 32 S 30 60 S 33 34 S 33 35
       S 33 56 S 36 37 S 37 38 S 37 39 S 37 43 S 39 40 S 39 41 S 39 42
       S 43 44 S 43 45 S 43 46 S 48 49 S 48 50 S 48 51 S 52 53 S 52 54
       S 52 55 S 56 57 S 56 58 S 56 59 S 60 61 S 60 62 S 60 63 S 64 65
       S 64 66 S 64 67 S 68 70 D 68 71 D 69 72 S 69 73 S 69 78 D 70 73
       S 70 74 S 71 72 S 71 75 S 72 76 S 73 77 S 79 80 S 79 81 END
END
```

```
STR12
  LONE 1 1 3 4 4 4 7 1 16 1 36 1 80 3 81 2 END
  BOND D 1 2 D 1 6 S 2 5 S 2 16 S 6 7 S 6 10 S 7 8 S 7 25 S 8 9 S 8 48
       S 8 52 S 9 10 S 9 11 S 9 12 S 10 64 S 10 68 D 13 14 S 13 18 S 13 19
       S 14 15 S 14 20 S 15 16 D 15 36 S 16 17 D 17 18 S 17 21 S 18 79
       S 22 23 D 22 27 S 22 47 D 23 24 S 23 28 S 24 25 S 24 33 D 25 26
       S 26 27 S 26 30 S 27 29 S 30 31 S 30 32 S 30 60 S 33 34 S 33 35
       S 33 56 S 36 37 S 37 38 S 37 39 S 37 43 S 39 40 S 39 41 S 39 42
       S 43 44 S 43 45 S 43 46 S 48 49 S 48 50 S 48 51 S 52 53 S 52 54
       S 52 55 S 56 57 S 56 58 S 56 59 S 60 61 S 60 62 S 60 63 S 64 65
       S 64 66 S 64 67 S 68 70 D 68 71 D 69 72 S 69 73 S 69 78 D 70 73
       S 70 74 S 71 72 S 71 75 S 72 76 S 73 77 S 79 80 D 79 81 END
END
STR13
  LONE 1 2 3 3 4 3 14 1 36 1 79 1 80 3 81 3 END
  BOND S 1 2 S 1 3 S 1 4 S 1 6 S 2 5 S 2 16 D 6 7 S 6 10 S 7 8 S 7 25
       S 8 9 S 8 48 S 8 52 S 9 10 S 9 11 S 9 12 S 10 64 S 10 68 S 13 14
       D 13 18 S 13 19 S 14 15 S 14 20 S 15 16 D 15 36 D 16 17 S 17 18
       S 17 21 S 18 79 S 22 23 D 22 27 S 22 47 D 23 24 S 23 28 S 24 25
       S 24 33 D 25 26 S 26 27 S 26 30 S 27 29 S 30 31 S 30 32 S 30 60
       S 33 34 S 33 35 S 33 56 S 36 37 S 37 38 S 37 39 S 37 43 S 39 40
       S 39 41 S 39 42 S 43 44 S 43 45 S 43 46 S 48 49 S 48 50 S 48 51
       S 52 53 S 52 54 S 52 55 S 56 57 S 56 58 S 56 59 S 60 61 S 60 62
       S 60 63 S 64 65 S 64 66 S 64 67 S 68 70 D 68 71 D 69 72 S 69 73
       S 69 78 D 70 73 S 70 74 S 71 72 S 71 75 S 72 76 S 73 77 S 79 80
       S 79 81 END
END
STR14
  LONE 1 2 3 4 4 4 36 2 80 3 81 2 END
  BOND D 1 2 S 1 6 S 2 5 S 2 16 D 6 7 S 6 10 S 7 8 S 7 25 S 8 9 S 8 48
       S 8 52 S 9 10 S 9 11 S 9 12 S 10 64 S 10 68 D 13 14 S 13 18 S 13 19
       S 14 15 S 14 20 D 15 16 S 15 36 S 16 17 D 17 18 S 17 21 S 18 79
       S 22 23 D 22 27 S 22 47 D 23 24 S 23 28 S 24 25 S 24 33 D 25 26
       S 26 27 S 26 30 S 27 29 S 30 31 S 30 32 S 30 60 S 33 34 S 33 35
       S 33 56 S 36 37 S 37 38 S 37 39 S 37 43 S 39 40 S 39 41 S 39 42
       S 43 44 S 43 45 S 43 46 S 48 49 S 48 50 S 48 51 S 52 53 S 52 54
       S 52 55 S 56 57 S 56 58 S 56 59 S 60 61 S 60 62 S 60 63 S 64 65
       S 64 66 S 64 67 S 68 70 D 68 71 D 69 72 S 69 73 S 69 78 D 70 73
       S 70 74 S 71 72 S 71 75 S 72 76 S 73 77 S 79 80 D 79 81 END
END
STR15
  LONE 1 3 3 4 4 3 36 2 79 1 80 3 81 3 END
  BOND S 1 2 S 1 4 S 2 5 S 2 16 D 6 7 S 6 10 S 7 8 S 7 25 S 8 9 S 8 48
       S 8 52 S 9 10 S 9 11 S 9 12 S 10 64 S 10 68 S 13 14 D 13 18 S 13 19
       D 14 15 S 14 20 S 15 16 S 15 36 D 16 17 S 17 18 S 17 21 S 18 79
       S 22 23 D 22 27 S 22 47 D 23 24 S 23 28 S 24 25 S 24 33 D 25 26
       S 26 27 S 26 30 S 27 29 S 30 31 S 30 32 S 30 60 S 33 34 S 33 35
       S 33 56 S 36 37 S 37 38 S 37 39 S 37 43 S 39 40 S 39 41 S 39 42
       S 43 44 S 43 45 S 43 46 S 48 49 S 48 50 S 48 51 S 52 53 S 52 54
       S 52 55 S 56 57 S 56 58 S 56 59 S 60 61 S 60 62 S 60 63 S 64 65
       S 64 66 S 64 67 S 68 70 D 68 71 D 69 72 S 69 73 S 69 78 D 70 73
       S 70 74 S 71 72 S 71 75 S 72 76 S 73 77 S 79 80 S 79 81 END
END
STR16
  LONE 1 3 3 3 4 4 36 2 79 1 80 3 81 3 END
  BOND S 1 2 S 1 3 S 2 5 S 2 16 D 6 7 S 6 10 S 7 8 S 7 25 S 8 9 S 8 48
       S 8 52 S 9 10 S 9 11 S 9 12 S 10 64 S 10 68 S 13 14 D 13 18 S 13 19
       D 14 15 S 14 20 S 15 16 S 15 36 D 16 17 S 17 18 S 17 21 S 18 79
       S 22 23 D 22 27 S 22 47 D 23 24 S 23 28 S 24 25 S 24 33 D 25 26
       S 26 27 S 26 30 S 27 29 S 30 31 S 30 32 S 30 60 S 33 34 S 33 35
       S 33 56 S 36 37 S 37 38 S 37 39 S 37 43 S 39 40 S 39 41 S 39 42
       S 43 44 S 43 45 S 43 46 S 48 49 S 48 50 S 48 51 S 52 53 S 52 54
       S 52 55 S 56 57 S 56 58 S 56 59 S 60 61 S 60 62 S 60 63 S 64 65
       S 64 66 S 64 67 S 68 70 D 68 71 D 69 72 S 69 73 S 69 78 D 70 73
       S 70 74 S 71 72 S 71 75 S 72 76 S 73 77 S 79 80 S 79 81 END
END
STR17
  LONE 1 3 3 4 4 3 36 2 79 1 80 3 81 3 END
  BOND S 1 2 S 1 4 S 2 5 S 2 16 D 6 7 S 6 10 S 7 8 S 7 25 S 8 9 S 8 48
       S 8 52 S 9 10 S 9 11 S 9 12 S 10 64 S 10 68 S 13 14 D 13 18 S 13 19
       D 14 15 S 14 20 S 15 16 S 15 36 D 16 17 S 17 18 S 17 21 S 18 79
       S 22 23 D 22 27 S 22 47 D 23 24 S 23 28 S 24 25 S 24 33 D 25 26
       S 26 27 S 26 30 S 27 29 S 30 31 S 30 32 S 30 60 S 33 34 S 33 35
```

```
          S 33 56 S 36 37 S 37 38 S 37 39 S 37 43 S 39 40 S 39 41 S 39 42
          S 43 44 S 43 45 S 43 46 S 48 49 S 48 50 S 48 51 S 52 53 S 52 54
          S 52 55 S 56 57 S 56 58 S 56 59 S 60 61 S 60 62 S 60 63 S 64 65
          S 64 66 S 64 67 S 68 70 D 68 71 D 69 72 S 69 73 S 69 78 D 70 73
          S 70 74 S 71 72 S 71 75 S 72 76 S 73 77 S 79 80 S 79 81 END
   END
   STR18
     LONE 1 3 3 3 4 4 36 2 79 1 80 3 81 3 END
     BOND S 1 2 S 1 3 S 2 5 S 2 16 D 6 7 S 6 10 S 7 8 S 7 25 S 8 9 S 8 48
          S 8 52 S 9 10 S 9 11 S 9 12 S 10 64 S 10 68 S 13 14 D 13 18 S 13 19
          D 14 15 S 14 20 S 15 16 S 15 36 D 16 17 S 17 18 S 17 21 S 18 79
          S 22 23 D 22 27 S 22 47 D 23 24 S 23 28 S 24 25 S 24 33 D 25 26
          S 26 27 S 26 30 S 27 29 S 30 31 S 30 32 S 30 60 S 33 34 S 33 35
          S 33 56 S 36 37 S 37 38 S 37 39 S 37 43 S 39 40 S 39 41 S 39 42
          S 43 44 S 43 45 S 43 46 S 48 49 S 48 50 S 48 51 S 52 53 S 52 54
          S 52 55 S 56 57 S 56 58 S 56 59 S 60 61 S 60 62 S 60 63 S 64 65
          S 64 66 S 64 67 S 68 70 D 68 71 D 69 72 S 69 73 S 69 78 D 70 73
          S 70 74 S 71 72 S 71 75 S 72 76 S 73 77 S 79 80 S 79 81 END
   END
   STR19
     LONE 1 3 3 3 4 3 6 1 36 2 79 1 80 3 81 3 END
     BOND S 1 3 S 1 4 S 2 5 S 2 16 D 6 7 S 6 10 S 7 8 S 7 25 S 8 9 S 8 48
          S 8 52 S 9 10 S 9 11 S 9 12 S 10 64 S 10 68 S 13 14 D 13 18 S 13 19
          D 14 15 S 14 20 S 15 16 S 15 36 D 16 17 S 17 18 S 17 21 S 18 79
          S 22 23 D 22 27 S 22 47 D 23 24 S 23 28 S 24 25 S 24 33 D 25 26
          S 26 27 S 26 30 S 27 29 S 30 31 S 30 32 S 30 60 S 33 34 S 33 35
          S 33 56 S 36 37 S 37 38 S 37 39 S 37 43 S 39 40 S 39 41 S 39 42
          S 43 44 S 43 45 S 43 46 S 48 49 S 48 50 S 48 51 S 52 53 S 52 54
          S 52 55 S 56 57 S 56 58 S 56 59 S 60 61 S 60 62 S 60 63 S 64 65
          S 64 66 S 64 67 S 68 70 D 68 71 D 69 72 S 69 73 S 69 78 D 70 73
          S 70 74 S 71 72 S 71 75 S 72 76 S 73 77 S 79 80 S 79 81 END
   END
   STR20
     LONE 1 3 3 3 4 4 36 2 79 1 80 3 81 3 END
     BOND S 1 2 S 1 3 S 2 5 S 2 16 D 6 7 S 6 10 S 7 8 S 7 25 S 8 9 S 8 48
          S 8 52 S 9 10 S 9 11 S 9 12 S 10 64 S 10 68 S 13 14 D 13 18 S 13 19
          D 14 15 S 14 20 S 15 16 S 15 36 D 16 17 S 17 18 S 17 21 S 18 79
          S 22 23 D 22 27 S 22 47 D 23 24 S 23 28 S 24 25 S 24 33 D 25 26
          S 26 27 S 26 30 S 27 29 S 30 31 S 30 32 S 30 60 S 33 34 S 33 35
          S 33 56 S 36 37 S 37 38 S 37 39 S 37 43 S 39 40 S 39 41 S 39 42
          S 43 44 S 43 45 S 43 46 S 48 49 S 48 50 S 48 51 S 52 53 S 52 54
          S 52 55 S 56 57 S 56 58 S 56 59 S 60 61 S 60 62 S 60 63 S 64 65
          S 64 66 S 64 67 S 68 70 D 68 71 D 69 72 S 69 73 S 69 78 D 70 73
          S 70 74 S 71 72 S 71 75 S 72 76 S 73 77 S 79 80 S 79 81 END
   END
   STR21
     LONE 1 2 3 3 4 3 36 2 79 1 80 3 81 3 END
     BOND S 1 2 S 1 3 S 1 4 S 1 6 S 2 5 S 2 16 D 6 7 S 6 10 S 7 8 S 7 25
          S 8 9 S 8 48 S 8 52 S 9 10 S 9 11 S 9 12 S 10 64 S 10 68 S 13 14
          D 13 18 S 13 19 D 14 15 S 14 20 S 15 16 S 15 36 D 16 17 S 17 18
          S 17 21 S 18 79 S 22 23 D 22 27 S 22 47 D 23 24 S 23 28 S 24 25
          S 24 33 D 25 26 S 26 27 S 26 30 S 27 29 S 30 31 S 30 32 S 30 60
          S 33 34 S 33 35 S 33 56 S 36 37 S 37 38 S 37 39 S 37 43 S 39 40
          S 39 41 S 39 42 S 43 44 S 43 45 S 43 46 S 48 49 S 48 50 S 48 51
          S 52 53 S 52 54 S 52 55 S 56 57 S 56 58 S 56 59 S 60 61 S 60 62
          S 60 63 S 64 65 S 64 66 S 64 67 S 68 70 D 68 71 D 69 72 S 69 73
          S 69 78 D 70 73 S 70 74 S 71 72 S 71 75 S 72 76 S 73 77 S 79 80
          S 79 81 END
   END
   STR22
     LONE 1 3 3 3 4 3 6 1 36 2 79 1 80 3 81 3 END
     BOND S 1 3 S 1 4 S 2 5 S 2 16 D 6 7 S 6 10 S 7 8 S 7 25 S 8 9 S 8 48
          S 8 52 S 9 10 S 9 11 S 9 12 S 10 64 S 10 68 S 13 14 D 13 18 S 13 19
          D 14 15 S 14 20 S 15 16 S 15 36 D 16 17 S 17 18 S 17 21 S 18 79
          S 22 23 D 22 27 S 22 47 D 23 24 S 23 28 S 24 25 S 24 33 D 25 26
          S 26 27 S 26 30 S 27 29 S 30 31 S 30 32 S 30 60 S 33 34 S 33 35
          S 33 56 S 36 37 S 37 38 S 37 39 S 37 43 S 39 40 S 39 41 S 39 42
          S 43 44 S 43 45 S 43 46 S 48 49 S 48 50 S 48 51 S 52 53 S 52 54
          S 52 55 S 56 57 S 56 58 S 56 59 S 60 61 S 60 62 S 60 63 S 64 65
          S 64 66 S 64 67 S 68 70 D 68 71 D 69 72 S 69 73 S 69 78 D 70 73
          S 70 74 S 71 72 S 71 75 S 72 76 S 73 77 S 79 80 S 79 81 END
   END
 $END
```

S2.3 Computational Study of the Initiation Pathway

S2.3.1 Interchange Pathway for HII

To locate the interchange transition state, we commenced from the DFT-optimized geometry reported by Percy and coworkers for ethyl vinyl ether (EVE) as substrate,[3] replacing the ethyl substituent with a *t*-butyl group. The optimized geometry **TS-IC-HII** is shown in Figure S19. This transition state is 9.6 kcal/mol less stable than the rate-determining transition state for the dissociative pathway (**TS2-3'-HII**; Scheme S1 and Figure S20), and 31.9 kcal/mol above the precursor. We also obtained a more stable conformation of the interchange transition state (**TS-IC'-HII,** 29.5 kcal/mol above the precursor): Figure S19 provides a 3D model of the optimized geometry. The main difference between the two geometries lies in the orientation of the *t*-butyl group. As well, however, the low-energy conformer is significantly less stable (by 7.2 kcal/mol) than **TS2-3'-HII**, strongly suggesting that initiation with tBuVE follows a dissociative mechanism.

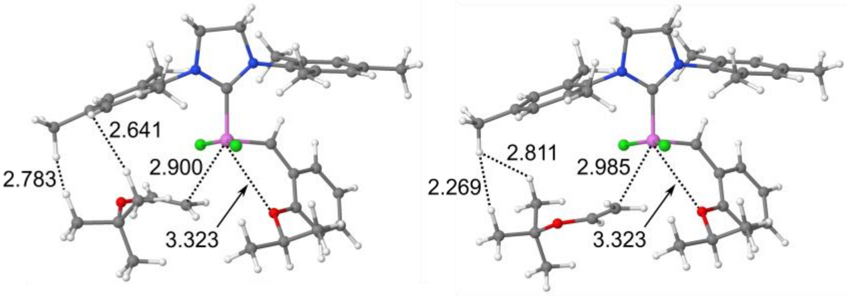

TS-IC-HII (31.9 kcal/mol) **TS-IC'-HII** (29.5 kcal/mol)

Figure S19. DFT-optimized geometries of two interchange-type transition states for alkene coordination to **HII**. The two transition states differ in the conformation of the tBuVE moiety. Ru: pink; Cl: green; C: gray; N: blue; H: white. Selected interatomic distances are given in Angstroms (Å). Free energies in chloroform relative to precursor **1** are given in parentheses.

S2.3.1 Complete Pathway for HII

Scheme S1. Initiation Pathway of HII, HC1Ph and nG-C1Ph with tBuVE.

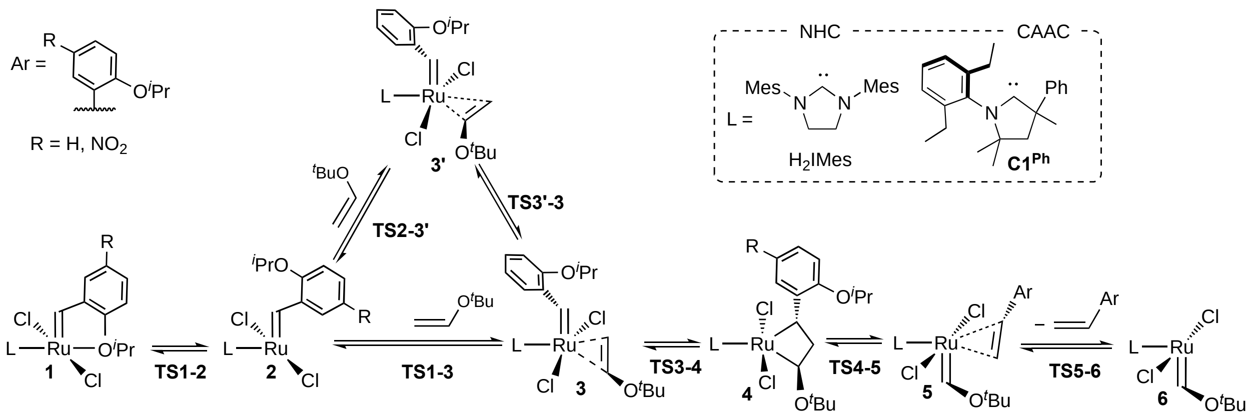

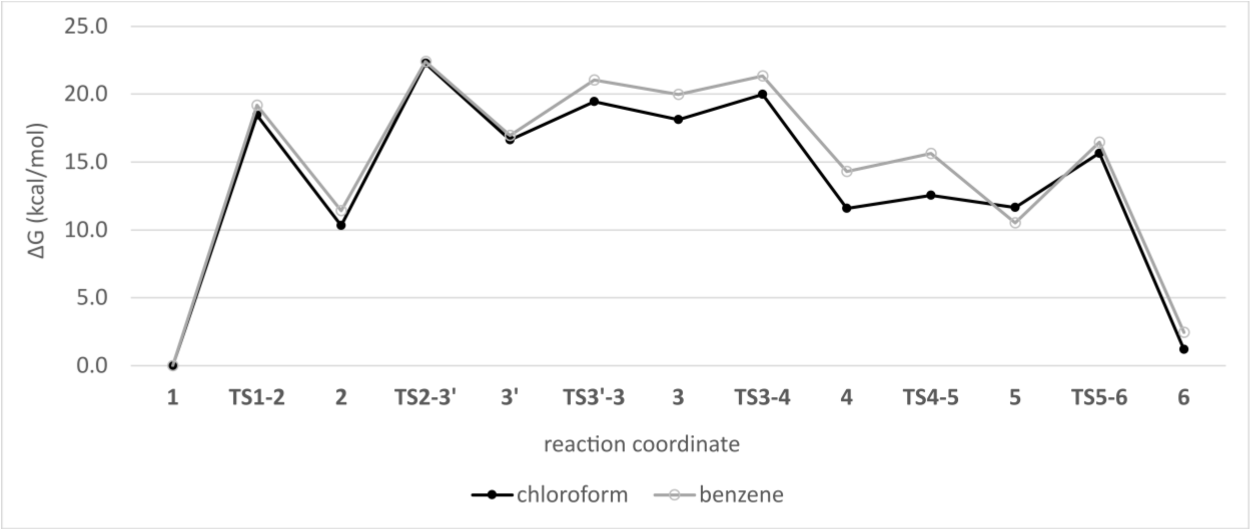

Figure S20. Calculated Gibbs free energies for the initiation of **HII** with tBuVE in $CHCl_3$ (black) and C_6H_6 (gray). Energies are relative to precursor **1**.

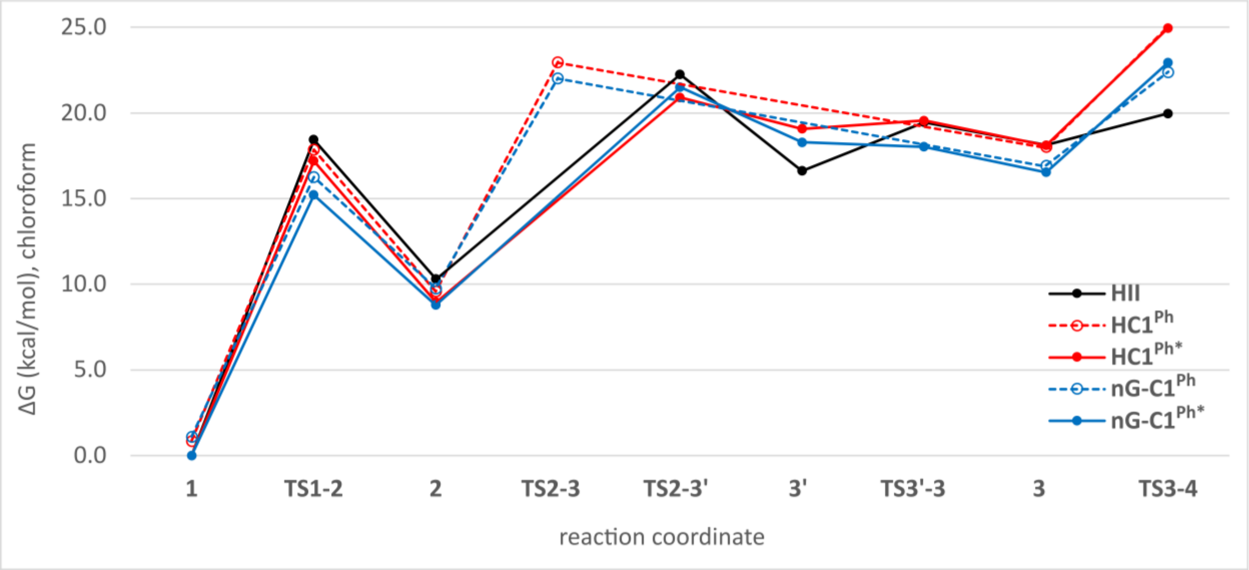

Figure S21. Calculated Gibbs free energies of the most favorable initiation pathways of **HII**, **HC1^{Ph}** and **nG-C1^{Ph}** with tBuVE in $CHCl_3$. Energies are given relative to the most stable rotamer of **1**. Within the **C1^{Ph}** species, the label (*) denotes the rotamer in which the alkylidene is syn to CMePh; that in which it is syn to N-Ar bears no asterisk (see Figure S9).

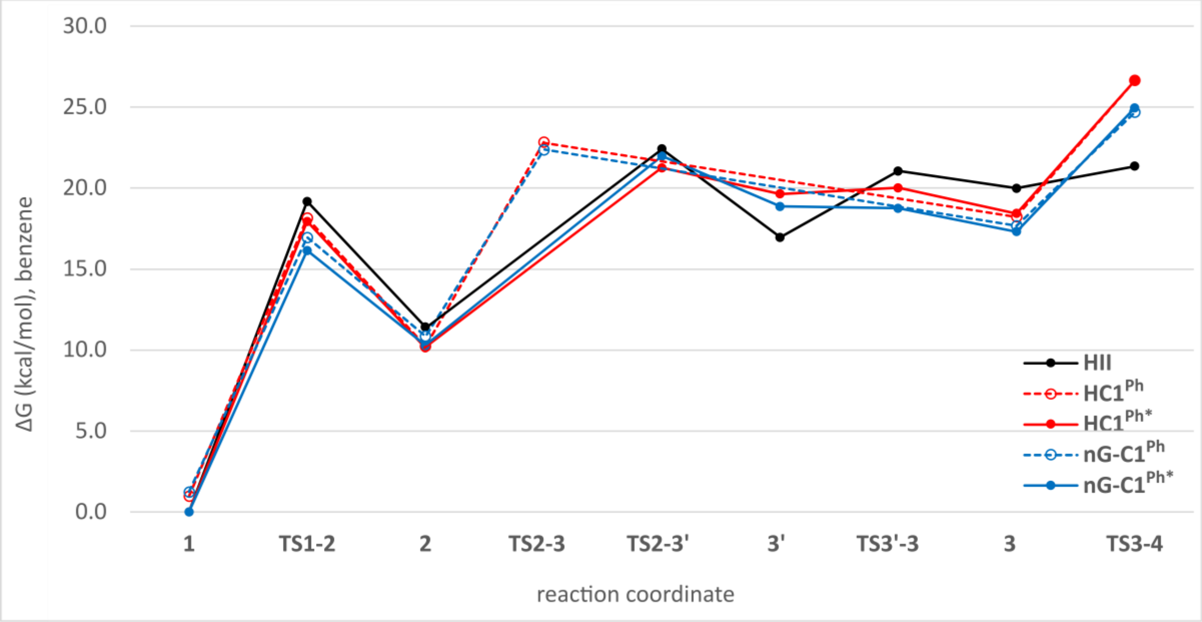

Figure S22. Calculated Gibbs free energies of the most favorable initiation pathways of **HII**, **HC1^{Ph}** and **nG-C1^{Ph}** with tBuVE as in Figure S21, but in C_6H_6.

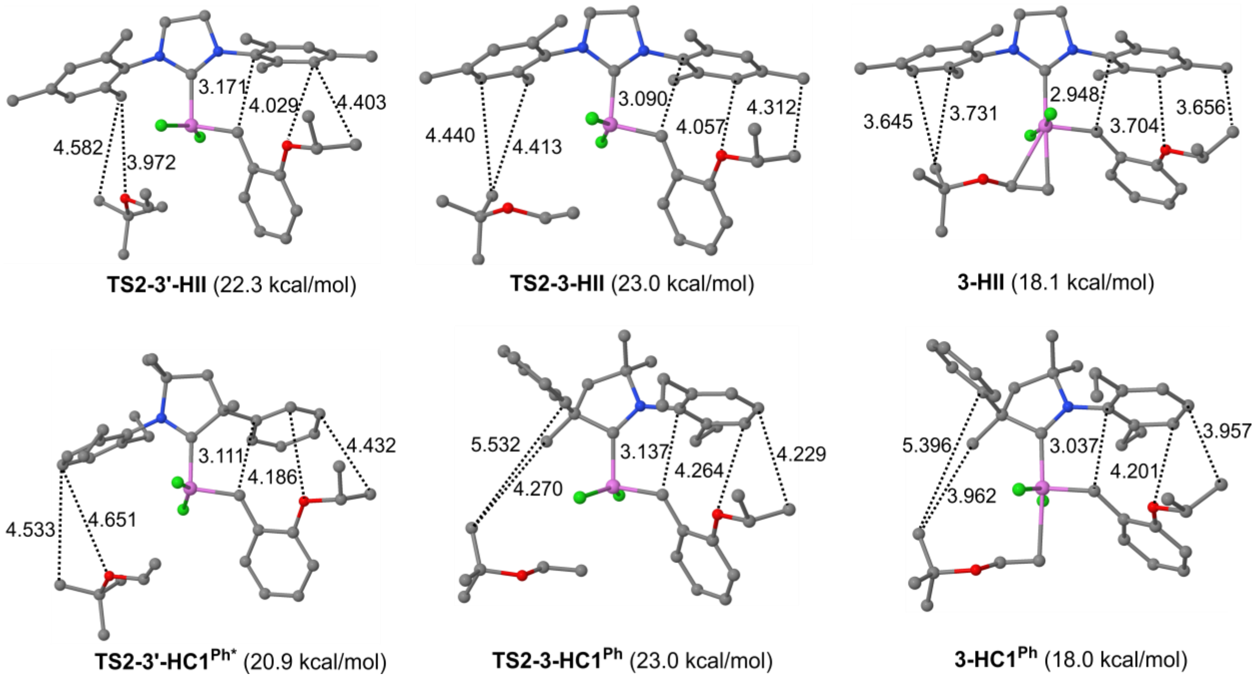

Figure S23. Selected shortest interatomic distances in Angstroms (Å) between the carbene ligand and the alkylidene and tBuVE moieties in the optimized geometries of **TS2-3'**, **TS2-3**, and **3** for **HII** (top) and **HC1$^{\mathbf{Ph}}$** (bottom). For clarity, hydrogen atoms are omitted.

S2.3.3 Effects of Solvent Coordination on the Initiation Rate

To test whether solvent binding can affect the initiation rate by generating low-energy states (thermodynamic sinks), we explored chloroform adducts of **HII** and **HC1$^{\mathbf{Ph}}$** (Figure S24). Formation of stable solvent complexes was investigated for the electronically unsaturated 14-electron complex **2**. Many different coordination modes were attempted for benzene, but none led to a stable complex.

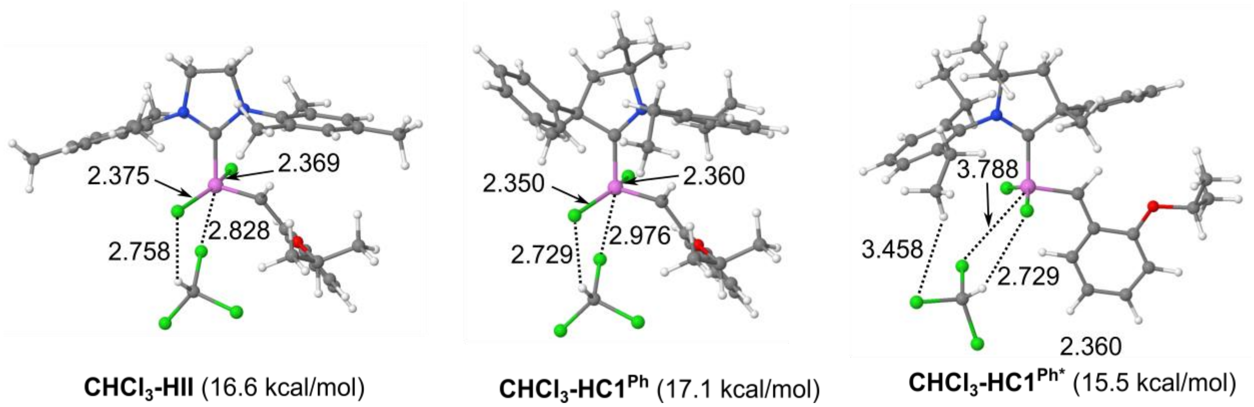

Figure S24. DFT-optimized geometries of the $CHCl_3$-complexes $CHCl_3$-**HII**, $CHCl_3$-**HC1$^{\mathbf{Ph}}$**, and $CHCl_3$-**HC1$^{\mathbf{Ph*}}$**. Ru: pink; Cl: green; C: gray; N: blue; H: white. Selected interatomic distances are given in Angstroms (Å). Values in parentheses are free energies (in chloroform) relative to **1**.

The relatively long interatomic distances between chloroform and the ruthenium center (2.828–3.788 Å), along with the small difference between the two Ru–Cl bond distances, indicate weak interactions between ruthenium and chloroform (Figure S24).

Taking into account the experimental concentrations ([catalyst] = 0.025 M; [chloroform] = 11.64 M), as described in the Computational Methods section, the Ru–chloroform species are 15.5–17.1 kcal/mol less stable than the precursor complexes **1**, and 6.3-7.5 kcal/mol less stable than the 14-electron complexes **2**. We infer that Ru–chloroform complexes are likely to form, but their concentrations will be negligible compared to the naked 14-electron complexes **2**, and the presence of solvent is thus not expected to hamper reaction of **2** with tBuVE. To significantly affect the rate of initiation, off-cycle species such as these solvent complexes would have to be more stable than, or at least of comparable stability to, the precursor **1**.

S2.3.4 All DFT-Calculated Absolute and Relative Energies

Table S3. Calculated Energies and Standard-State Gibbs Free Energies. Stationary points of unfavorable pathways are highlighted in grey.

Molecular Model ID	$E^{CHCl_3}_{PBE}$ [a.u.]	$G^{CHCl_3\,298.15K}_{PBE}$ [a.u.]	$E^{CHCl_3}_{PBE\text{-}D3M(BJ)}$ [a.u.]	$E^{C_6H_6}_{PBE\text{-}D3M(BJ)}$ [a.u.]	$G^{CHCl_3\,298.15K\,[1M]}_{PBE\text{-}D3M(BJ)}$ [a.u.]	$G^{C_6H_6\,298.15K\,[1M]}_{PBE\text{-}D3M(BJ)}$ [a.u.]	$\Delta G^{CHCl_3\,298.15K\,[1M]}_{PBE\text{-}D3M(BJ)}$ [kcal/mol]	$\Delta G^{C_6H_6\,298.15K\,[1M]}_{PBE\text{-}D3M(BJ)}$ [kcal/mol]
tBuVE	-310.661481	0.128379	-310.806675	-310.803928	-310.675934	-310.673187		
2-isopropoxystyrene	-502.182532	0.175242	-502.398275	-502.394458	-502.223496	-502.219680		
**1_HII**	-2401.997965	0.517892	-2402.689068	-2402.672830	-2402.171640	-2402.155402	0.0	0.0
**1_HC1^{Ph}**	-2425.184795	0.569029	-2425.899609	-2425.885579	-2425.331043	-2425.317014	0.9	1.0
**1_HC1^{Ph*}**	-2425.186023	0.567150	-2425.899107	-2425.885240	-2425.332421	-2425.318554	0.0	0.0
**1_nG-C1^{Ph}**	-2629.532711	0.567946	-2630.330593	-2630.315216	-2629.763111	-2629.747734	1.1	1.2
**1_nG-C1^{Ph*}**	-2629.533831	0.565640	-2630.330044	-2630.314882	-2629.764868	-2629.749706	0.0	0.0
**TS-IC'_HII**	-2712.627696	0.669074	-2713.469178	-2713.450111	-2712.800568	-2712.781501	29.5	29.5
**TS-IC_HII**	-2712.624003	0.672191	-2713.468512	-2713.449789	-2712.796784	-2712.778062	31.9	31.7
**TS1-2_HII**	-2401.972710	0.517812	-2402.659601	-2402.642195	-2402.142253	-2402.124847	18.4	19.2
**T1_2_HC1^{Ph}**	-2425.158283	0.566277	-2425.869767	-2425.855443	-2425.303954	-2425.289630	17.9	18.2
**TS1_2_HC1^{Ph*}**	-2425.162296	0.566090	-2425.870637	-2425.855598	-2425.305011	-2425.289971	17.2	17.9
**TS1_2_nG-C1^{Ph}**	-2629.508439	0.565248	-2630.303727	-2630.287456	-2629.738943	-2629.722672	16.3	17.0
**TS1_2_nG-C1^{Ph*}**	-2629.511949	0.564130	-2630.304282	-2630.287631	-2629.740615	-2629.723965	15.2	16.2
**2_HII**	-2401.982513	0.515693	-2402.670435	-2402.652450	-2402.155205	-2402.137221	10.3	11.4
**2_HC1^{Ph}**	-2425.169490	0.564488	-2425.881176	-2425.866278	-2425.317151	-2425.302254	9.6	10.2
**2_HC1^{Ph*}**	-2425.173789	0.564939	-2425.882597	-2425.866901	-2425.318122	-2425.302426	9.0	10.1
**2_nG-C1^{Ph}**	-2629.518025	0.564065	-2630.312910	-2630.296024	-2629.749308	-2629.732423	9.8	10.8
**2_nG-C1^{Ph*}**	-2629.522402	0.563730	-2630.314133	-2630.296604	-2629.750867	-2629.733338	8.8	10.3
**TS2-3'_HII**	-2712.636712	0.666790	-2713.478425	-2713.459191	-2712.812099	-2712.792865	22.3	22.4
**T2_3'_HC1^{Ph}**	-2735.822486	0.716264	-2736.686146	-2736.669984	-2735.970346	-2735.954184	23.9	23.6

**TS2_3'_HC1^{Ph}***	-2735.826581	0.714631	-2736.689208	-2736.672039	-2735.975041	-2735.957871	20.9	21.3
**TS2_3'_nG-C1^{Ph}**	-2940.174259	0.715475	-2941.119058	-2941.101678	-2940.404047	-2940.386667	23.1	22.7
**TS2_3'_nG-C1^{Ph}***	-2940.178202	0.716747	-2941.122786	-2941.104148	-2940.406503	-2940.387865	21.5	22.0
**TS2-3_HII**	-2712.635382	0.664954	-2713.475446	-2713.455175	-2712.810956	-2712.790684	23.0	23.8
**T2_3_HC1^{Ph}**	-2735.823228	0.716524	-2736.687806	-2736.671409	-2735.971746	-2735.955348	23.0	22.8
**TS2_3_nG-C1^{Ph}**	-2940.172674	0.714914	-2941.120133	-2941.101693	-2940.405683	-2940.387243	22.0	22.4
**3'_HII**	-2712.645558	0.672223	-2713.492839	-2713.473330	-2712.82108	-2712.801571	16.6	17.0
**3'_HC1^{Ph}***	-2735.828884	0.717135	-2736.694637	-2736.677117	-2735.977966	-2735.960446	19.1	19.6
**3'_nG-C1^{Ph}***	-2940.181495	0.717582	-2941.128764	-2941.109940	-2940.411646	-2940.392821	18.3	18.9
**TS3'-3_HII**	-2712.646554	0.670329	-2713.486417	-2713.464900	-2712.816552	-2712.795035	19.5	21.1
**TS3'_3_HC1^{Ph}**	-2735.828875	0.718225	-2736.694953	-2736.677592	-2735.977192	-2735.959831	19.6	20.0
**TS3'_3_nG-C1^{Ph}**	-2940.179336	0.718661	-2941.130280	-2941.111193	-2940.412083	-2940.392996	18.0	18.8
**3_HII**	-2712.648836	0.677061	-2713.495300	-2713.473330	-2712.818703	-2712.796733	18.1	20.0
**3_HC1^{Ph}**	-2735.828505	0.718419	-2736.697577	-2736.680610	-2735.979622	-2735.962655	18.0	18.3
**3_HC1^{Ph}***	-2735.830300	0.720006	-2736.699040	-2736.681914	-2735.979498	-2735.962372	18.1	18.4
**3_nG-C1^{Ph}**	-2940.177838	0.717165	-2941.130510	-2941.111422	-2940.413809	-2940.394720	16.9	17.7
**3_nG-C1^{Ph}***	-2940.179764	0.718175	-2941.132152	-2941.113010	-2940.414441	-2940.395298	16.5	17.3
**TS3-4-HII**	-2712.646120	0.672314	-2713.487599	-2713.466412	-2712.815749	-2712.794561	20.0	21.4
**TS3-4-HC1^{Ph}**	-2735.824217	0.720290	-2736.688235	-2736.669069	-2735.968408	-2735.949243	25.1	26.7
**TS3-4-HC1^{Ph}***	-2735.828558	0.719713	-2736.687826	-2736.668533	-2735.968577	-2735.949284	25.0	26.6
**TS3-4-nG-C1^{Ph}**	-2940.174522	0.717367	-2941.122017	-2941.100432	-2940.405114	-2940.383529	22.4	24.7
**TS3-4-nG-C1^{Ph}***	-2940.178624	0.717912	-2941.121675	-2941.100568	-2940.404227	-2940.383120	23.0	25.0
**4_HII**	-2712.664954	0.673199	-2713.501846	-2713.478524	-2712.829111	-2712.805788	11.6	14.3
**TS4_5_HII**	-2712.662664	0.672804	-2713.499930	-2713.475999	-2712.827590	-2712.803659	12.5	15.6
**5_HII**	-2712.657397	0.666230	-2713.497838	-2713.477572	-2712.832072	-2712.811806	9.7	10.5
**TS5_6_HII**	-2712.652524	0.664034	-2713.486203	-2713.465921	-2712.822633	-2712.802351	15.7	16.5
**6_HII**	-2210.479164	0.468225	-2211.089935	-2211.072755	-2210.622174	-2210.604994	1.2	2.5
**$CHCl_3$-HII**	-3820.679030	0.527066	-3821.468908		-3820.942305		16.6	
**$CHCl_3$- HC1^{Ph}**	-3843.864823	0.575945	-3844.677802		-3844.102321		17.1	
**$CHCl_3$- HC1^{Ph}***	-3843.871291	0.571367	-3844.675759		-3844.104856		15.5	
chloroform	-1418.700717	-0.011472	-1418.791050		-1418.797190			

S3. References

(1) Glendening, E. D.; Badenhoop, J. K.; Reed, A. E.; Carpenter, J. E.; Bohmann, J. A.; Morales, C. M.; Karafiloglou, P.; Landis, C. R.; Weinhold, F. *NBO*, 7.0; Theoretical Chemistry Institute, University of Wisconsin: Madison, WI, 2018.

(2) (a) Glendening, E. D.; Weinhold, F. J. Natural Resonance Theory: I. General Formalism. *J. Comput. Chem.* **1998**, *19*, 593–609. (b) Glendening, E. D.; Weinhold, F. J. Natural Resonance Theory: II. Natural Bond Order and Valency. *J. Comput. Chem.* **1998**, *19*, 610–627. (c) Glendening, E. D.; Badenhoop, J. K.; Weinhold, F. J. Natural Resonance Theory: III. Chemical Applications. *J. Comput. Chem.* **1998**, *19*, 628–646.

(3) Ashworth, I. W.; Hillier, I. H.; Nelson, D. J.; Percy, J. M.; Vincent, M. A., Olefin Metathesis by Grubbs–Hoveyda Complexes: Computational and Experimental Studies of the Mechanism and Substrate-Dependent Kinetics. *ACS Catal.* **2013**, *3*, 1929-1939.
